# Genomic Epidemiology and Evolution of Rhinovirus in Western Washington State, 2021–2022

**DOI:** 10.1093/infdis/jiae347

**Published:** 2024-07-04

**Authors:** Stephanie Goya, Seffir T Wendm, Hong Xie, Tien V Nguyen, Sarina Barnes, Rohit R Shankar, Jaydee Sereewit, Kurtis Cruz, Ailyn C Pérez-Osorio, Margaret G Mills, Alexander L Greninger

**Affiliations:** Department of Laboratory Medicine and Pathology, University of Washington Medical Center, Seattle, Washington; Department of Laboratory Medicine and Pathology, University of Washington Medical Center, Seattle, Washington; Department of Laboratory Medicine and Pathology, University of Washington Medical Center, Seattle, Washington; Department of Laboratory Medicine and Pathology, University of Washington Medical Center, Seattle, Washington; Department of Laboratory Medicine and Pathology, University of Washington Medical Center, Seattle, Washington; Department of Laboratory Medicine and Pathology, University of Washington Medical Center, Seattle, Washington; Department of Laboratory Medicine and Pathology, University of Washington Medical Center, Seattle, Washington; Department of Laboratory Medicine and Pathology, University of Washington Medical Center, Seattle, Washington; Department of Laboratory Medicine and Pathology, University of Washington Medical Center, Seattle, Washington; Department of Laboratory Medicine and Pathology, University of Washington Medical Center, Seattle, Washington; Department of Laboratory Medicine and Pathology, University of Washington Medical Center, Seattle, Washington; Vaccine and Infectious Disease Division, Fred Hutchinson Cancer Research Center, Seattle, Washington

**Keywords:** rhinovirus, Washington, genomic epidemiology, VP1, viral evolution

## Abstract

**Background:**

Human rhinoviruses (RVs) primarily cause the common cold, but infection outcomes vary from subclinical to severe cases, including asthma exacerbations and fatal pneumonia in individuals who are immunocompromised. To date, therapeutic strategies have been hindered by the high diversity of serotypes. Global surveillance efforts have traditionally focused on sequencing VP1 or VP2/VP4 genetic regions, leaving gaps in our understanding of RV genomic diversity.

**Methods:**

We sequenced 1078 RV genomes from nasal swabs of symptomatic and asymptomatic individuals to explore viral evolution during 2 epidemiologically distinct periods in Washington State: when the COVID-19 pandemic affected the circulation of other seasonal respiratory viruses except for RV (February–July 2021) and when the seasonal viruses reemerged with the severe outbreak of respiratory syncytial virus and influenza (November–December 2022). We constructed maximum likelihood and BEAST phylodynamic trees to characterize intragenotype evolution.

**Results:**

We detected 99 of 168 known genotypes and observed intergenotypic recombination and genotype cluster swapping from 2021 to 2022. We found a significant association between the presence of symptoms and viral load but not with RV species or genotype. Phylodynamic trees, polyprotein selection pressure, and Shannon entropy revealed cocirculation of divergent clades within genotypes with high amino acid constraints throughout the polyprotein.

**Conclusions:**

Our study underscores the dynamic nature of RV genomic epidemiology within a localized geographic region, as >20% of existing genotypes within each RV species cocirculated each studied month. Our findings also emphasize the importance of investigating correlations between RV genotypes and serotypes to understand long-term immunity and cross-protection.

## Teaser

Human rhinovirus A–C species and their cocirculating genotypes show remarkable overall genetic diversity. Using the largest collection of newly sequenced rhinovirus genomes to date, we demonstrate extensive cocirculation of highly divergent clades within a genotype with highly constrained amino acid variability in the viral polyprotein.

Human rhinoviruses (RVs) are a primary etiology of the common cold, causing 8.9% to 19% of total acute upper respiratory infections [1–3]. A recent study of RV burden in New York City found that 23.5% of the individuals with acute upper respiratory infections associated with RV missed at least 1 day of work or school [4]. RV can also cause lower respiratory tract infections in patients who are immunocompromised, with a high mortality rate [5, 6], and it is a major contributor of exacerbations of asthma [7–9], cystic fibrosis [10], and chronic obstructive pulmonary disease [11, 12]. Asymptomatic RV infection is relatively common, with up to 4% to 6.6% of specimens taken from asymptomatic people testing positive for RV [2, 3]. While 100 RV serotypes were described by 1987 [13] and the first complete RV genome was published in 1984 [14], <500 complete RV genomes were available in public databases at the initiation of this study (January 2021), meaning that most RV genotypes had <5 genomes available. Despite its more widespread disease burden, RV genomic data pale in comparison to the 163 000 influenza virus genomes or 11 500 respiratory syncytial virus genomes currently available in GISAID or INSDC databases.

RVs are nonenveloped viruses with a single-stranded positive-sense genome of around 7300 nucleotides. The RV genome is organized into a single polyprotein that undergoes autocatalytically and protease-based cleavage to produce 4 capsid proteins (VP1–VP4) and 7 nonstructural proteins [15]. Global RV molecular epidemiology is based on the phylogenetic association of the VP1 genetic sequence, which classified 3 RV species (RV-A, RV-B, and RV-C) subclassified into genotypes (81 RV-A, 32 RV-B, and 55 RV-C genotypes) [16]. The cocirculation of many highly diverse genotypes, considered antigenically distinct, hampers the development of an effective, broadly reactive RV vaccine [17].

Here, using a set of >1000 newly generated RV genomes constituting more than half of publicly available RV genomes, we describe the epidemiology and evolution of RV from symptomatic and asymptomatic cases in the Puget Sound region (Washington State) during 2 distinct epidemiologic periods of the COVID-19 pandemic [18–22].

## MATERIALS AND METHODS

### Clinical Samples and Data Collection

Remnant SARS-CoV-2–negative nasal swabs collected from outpatients attending COVID-19 community testing sites at UW Medicine (University of Washington) were retrospectively randomly selected and screened for RV by reverse transcription quantitative polymerase chain reaction. Individuals declaring at least 1 respiratory symptom at the time of collection were defined as symptomatic. Reverse transcription quantitative polymerase chain reaction screening was performed in 2 periods: (1) samples from February to July 2021 were randomly selected, and 4 specimen pools were constructed with equal viral transport medium volumes prior to extraction, with a subset of positive pools screened individually (see Results, supplementary material); (2) samples collected in November and December 2022 were randomly selected and individually screened. Demographic and symptomatic information was collected from screening questionnaires during COVID-19 testing. This study was approved the UW Medicine Institutional Review Board with a consent waiver (STUDY00000408).

### Next-Generation Sequencing and Assembly of Viral Consensus Genomes

RNA from nasal swabs with an RV cycle threshold (Ct) <33 was sequenced by metagenomic next-generation sequencing as described previously [23] (supplementary material). Consensus genomes were called by using custom pipelines of mapping against references of all existing RV genomes from the International Committee on Taxonomy of Viruses as of March 2022.

### Sequence, Phylogenetic, and Statistical Analysis

Sequence, phylogenetic, and statistical analysis methods are described in the supplementary material. The R markdown code is available on GitHub (https://github.com/greninger-lab/HRV_epidemiology). FASTQ and RV consensus genome data are available in NCBI BioProject PRJNA1066815 (Supplementary Table 1).

## RESULTS

### Demographic and Clinical Characteristics of RV Infections

Our RV screening was based on a subset of SARS-CoV-2 negative nasal swab specimens collected at the UW COVID-19 community testing sites during February to July 2021 and November to December 2022. These 2 periods were epidemiologically distinct, as UW hospital-based surveillance data reported RV predominance since April 2020 until July 2021 with very low circulation of the other seasonal respiratory viruses in Washington State (Supplementary Figure 1). Since August 2021, seasonal respiratory viruses reemerged with unforeseen out-of-season outbreaks while the detection of influenza virus remained notably low. However, during the winter of 2022 to 2023, a significant outbreak of influenza virus and respiratory syncytial virus occurred, despite RV maintaining consistent circulation with monthly positive rates around 10%.

In 2021, we screened 33 528 samples in 8328 four-sample pools for RV and detected 3023 RV-positive pools (36.3%). Of these, 1296 positive pools were further analyzed to determine the individual positive samples. We obtained 1044 pools with 1 positive sample, 233 with 2, 18 with 3, and 1 with 4, resulting in 1568 individually confirmed RV-positive samples. Between November and December 2022, 802 samples were positive out of 10 656 individually screened samples. The RV-positive population consisted of 49% male persons and 57% between the ages of 18 and 65 years (Table 1).

**Table 1.** Demographic and Virologic Characteristic of Individuals Who Were RV Positive

	Total	Feb–Jul 2021	Nov–Dec 2022
RV RT-qPCR positive	3827^a^	3023^a^	804
RT-PCR Cycle threshold (Ct)	27.19 (22.78–31.72)^a^	26.41 (22.15–31.26)^a^	29.55 (25.92–32.58)
Individuals with demographic data	2372	1568	804
Male	1165 (49.1)	758 (48.3)	407 (50.6)
Age, y			
<5	405 (17.0)	264 (16.8)	141 (17.5)
5–18	671 (28.2)	579 (36.9)	92 (11.4)
19–65	1198 (50.5)	694 (44.3)	504 (62.7)
>65	98 (4.1)	31 (2.0)	67 (8.3)
Individuals with clinical data	2094	1519	575
Symptomatic	1453 (69.4)	1048 (69.0)	405 (70.4)
Asymptomatic	641 (30.6)	471 (31.0)	170 (29.6)

Data are presented as No. (%) or median (IQR). Percentages are shown regarding the number of individuals with available demographic or clinical data, which are a subset of those who tested positive for RV.

Abbreviations: RT-qPCR, reverse transcription quantitative polymerase chain reaction; RV, rhinovirus.

^a^RT-qPCR results from 2021 screening of 4-plex sample pooling (Supplementary Material).

**Figure 1.**
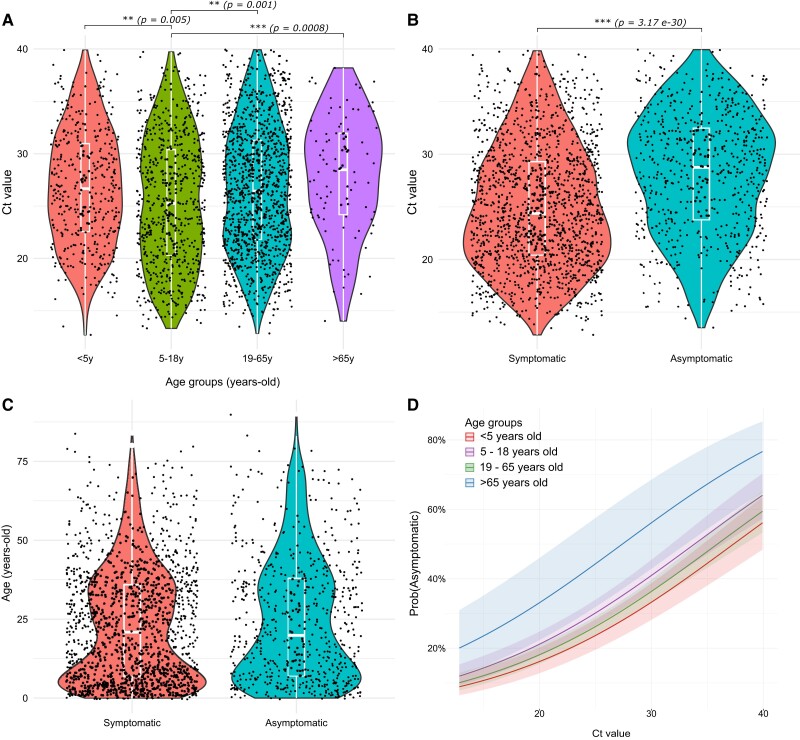
Rhinovirus infections and relationship with age group and symptom presence. The association of age, presence of respiratory symptoms, and Ct value was evaluated. Violin plots illustrate the distribution of cases for (*A*) Ct value and stratified age group, (*B*) Ct value and symptom presence, and (*C*) nonstratified age and symptom presence. Black dots represent individuals. Within the violin plot, a box plot shows the median, IQR, and 95% CI. A Kruskal-Wallis test was used to assess the association between variables and a Wilcoxon test for the age group pairwise analysis. ***P < .0001. **P < .001. *D*, Binomial logistic regression of symptom presence considering the effect of the Ct value and age group. The plot shows the probability model of an individual being asymptomatic for each age group. Shadows around the main line indicate 95% CI. Ct, cycle threshold.

**Figure 2.**
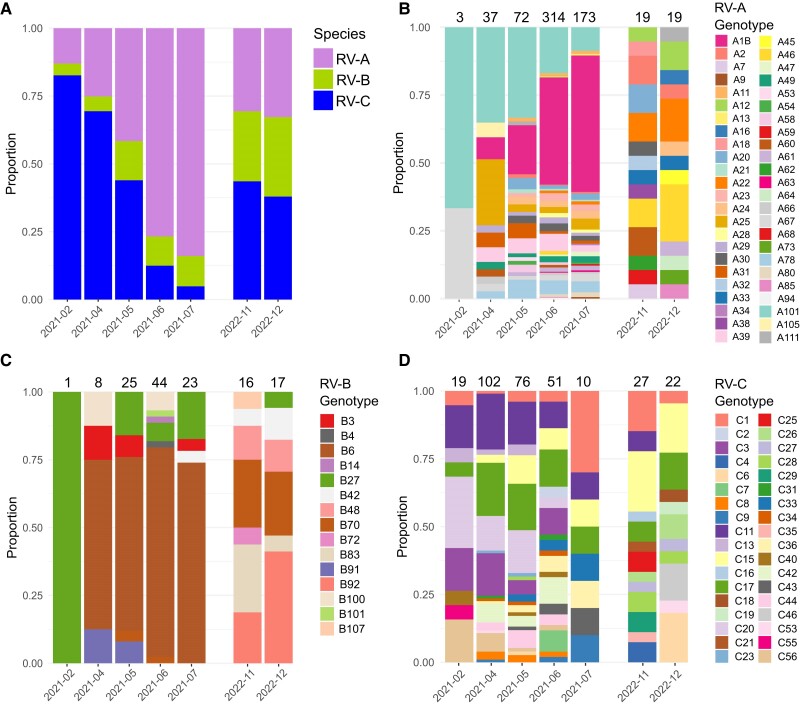
Abundance of rhinovirus (RV) species and genotypes within the sequenced genomes. The stacked bar plot shows the proportion of (*A*) RV species, (*B*) genotypes within RV-A, (*C*) genotypes within RV-B, and (*D*) genotypes within RV-C by studied month. For genotype bar plots, the total number of genomes per month is detailed above each column.

Among RV-positive cases with clinical data available, persons aged 5 to 18 years exhibited significantly lower Ct values as compared with those aged <5 or >18 years (Wilcoxon test, adjusted *P* < 5.0 × 10^−3^; Figure 1*A*). Symptomatic cases comprised 66% of the RV-positive population, with sore throat (51%), runny nose (36%), cough (31%), fever (18%), congestion (16%), and headache (8%) being the most common. Individuals reporting respiratory symptoms had lower Ct values than those who were asymptomatic (Kruskal-Wallis test, *P* = 3.17 × 10^−30^; Figure 1*B*). No association was found between the presence of symptoms and age as a continuous variable but rather when categorized into age groups (χ^2^ test, *P* = .007; Figure 1*C*). A binary logistic regression model was used to analyze the probability of being asymptomatic according to age group and Ct value (Figure 2*D*). The results confirm a positive relationship between the Ct value and the probability of being asymptomatic, which is stronger for older patients (model accuracy, 69.5%).

### Epidemiologic and Geographic Characterization of RV Species

We first characterized RV epidemiology at the species level. Metagenomic sequencing resulted in 1078 complete or near-complete RV genomes (958 from 2021 and 120 from 2022), consisting of 637 RV-A, 134 RV-B, and 307 RV-C. Seasonal analysis revealed an overall shift from RV-C to RV-A prevalence from February to July 2021 (Figure 2*A*). Given the higher prevalence of RV-C during November and December 2022, our data indicate a higher relative prevalence of RV-A in spring and summer, with RV-C circulating at higher levels in autumn and winter [24]. RV-B exhibited 10.5% of the total cases in 2021 and 27.5% in 2022.

RV species was not associated with either the presence of symptoms or sex but was associated with age (*G* test of independence, *P* = 9.21 × 10^−6^). Specifically, children <5 years old were more likely to be infected by RV-C than RV-A or RV-B (conditional maximum-likelihood estimate of odds ratio [95% CI]: RV-A vs RV-C, 0.41 [0.27–0.64]; RV-B vs RV-C, 0.25 [0.11–0.59]; Supplementary Table 2). Similarly, individuals aged >65 years were more likely to be infected by RV-C than RV-A (RV-A vs RV-C, 0.38 [0.15–0.95]). However, patients between 5 and 18 years old were more likely to be infected by RV-A than RV-C (RV-A vs RV-C, 1.75 [1.21–2.57]).

**Figure 3.**
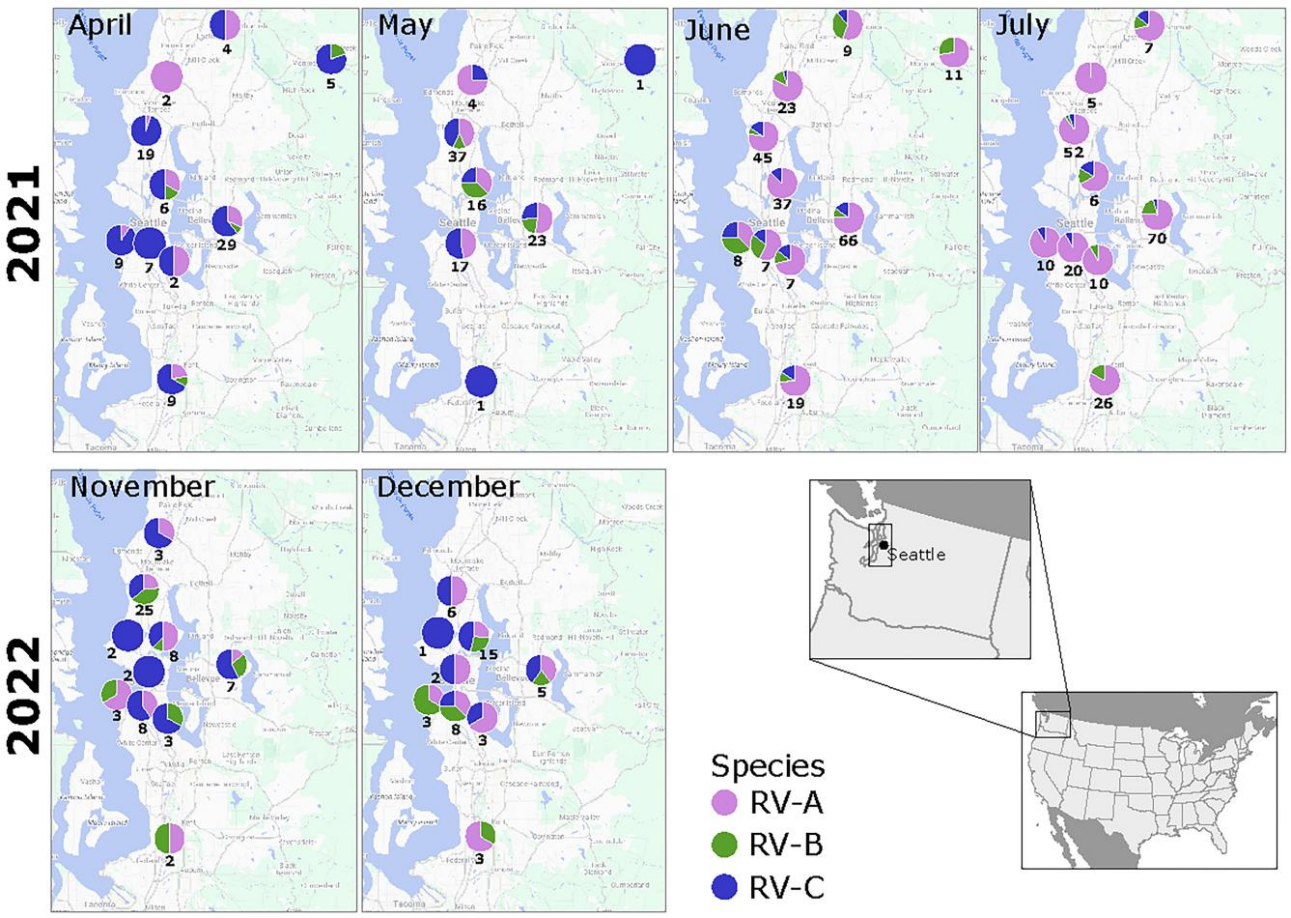
Geographic spread of rhinovirus (RV) species in Puget Sound region. For each studied month, the proportion of RV species (RV-A, pink; RV-B, green; RV-C, blue) is mapped according to the UW Medicine COVID-19 community testing sites where the individuals attended. The total number detected is shown below each pie plot. February 2021 is not shown due to the low number of cases available. A map at the bottom right highlights the geographic location of Puget Sound. UW, University of Washington.

We investigated if RV species distribution differed by location of COVID-19 community testing sites where specimens were collected. We found that the cocirculation, prevalence, and replacement from RV-A to RV-C were not geographically distinct during 2021, with no geographic distribution pattern observed in 2022, consistent with the overall seasonality (Figure 3).

### RV Genotype Diversity in the Puget Sound Region

We next characterized RV diversity at the genotype level. Data sets of 1321 RV-A, 321 RV-B, and 611 RV-C sequences were analyzed, comprising all whole genomes available in NCBI Virus as of October 2023, with VP1 nucleotide sequences to represent RV-A and RV-C genotypes without available genomes. To evaluate the continuity or reemergence of the genotype circulation in the Puget Sound region, 16 positive RV samples from the Seattle hospital-based respiratory virus surveillance from January to June 2023 were sequenced and included in the phylogenetic analyses. Within this data set containing all globally available RV genomes, 67% of sequences were collected in Washington State. All complete genomes were trimmed to the VP1 region to proceed with the globally established genotyping classification [16].

**Figure 4.**
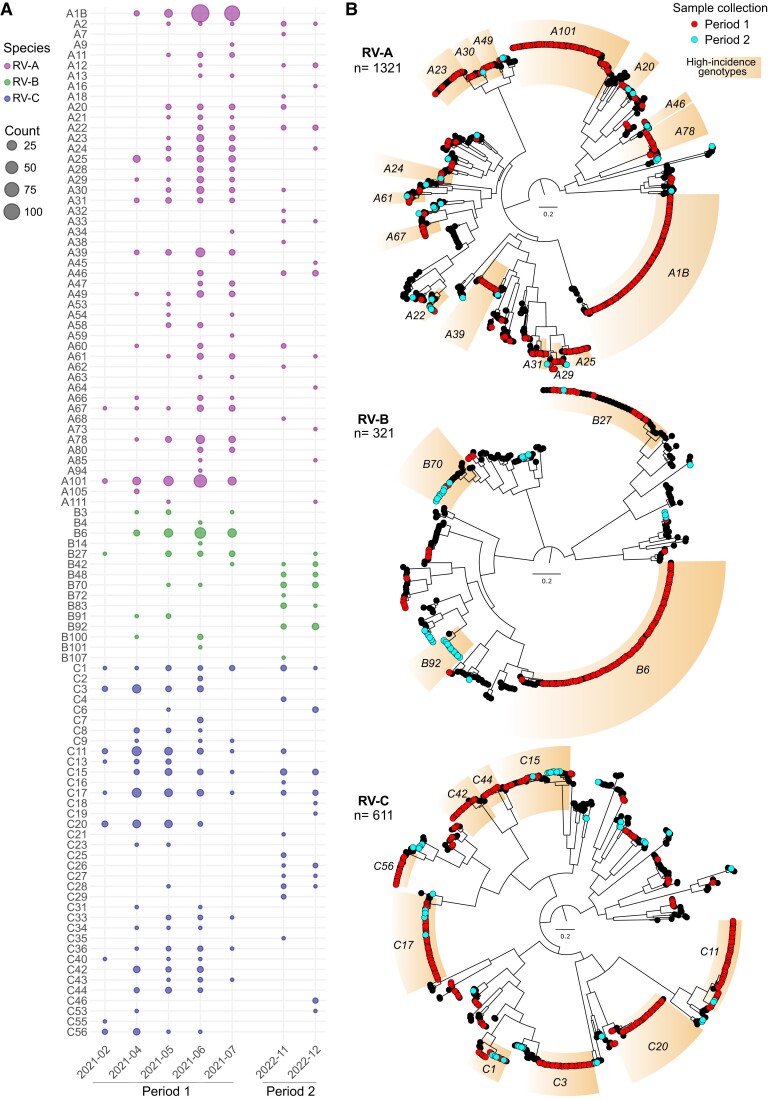
Genotyping of rhinovirus (RV) detected in Washington State in 2021 and 2022. *A*, Seasonality of RV genotypes detected within RV-A, RV-B, and RV-C. The bubble chart details the number of cases (by size of circle) for each genotype per month of the studied period. Circle color denotes the RV species. *B*, Maximum likelihood tree with the VP1 nucleotide sequence of the cases studied in this work and reference sequences. Tree tips of sequences from this study are denoted with a red or light blue circle if collected in period 1 or 2, respectively. The total number of sequences in the tree is informed below the rhinovirus species. High-frequency genotypes, defined as >1% of the cases within the RV species and >10 sequences available, are highlighted orange, including the name of the genotype in italics. Scale bar indicates substitution per site.

We detected 48 RV-A, 15 RV-B, and 36 RV-C genotypes in 2021 to 2022 (Figures 2*B*–2*D*, 4*A*, and 4*B*). We identified genotypes with a frequency >1% of RV species cases in any given month during the study period. We further analyzed high-frequency genotypes that comprised >10 genomes within our data set, including 16 RV-A, 4 RV-B, and 9 RV-C genotypes. High RV-C prevalence from February to April 2021 was associated with a stably even distribution of high-frequency genotypes, while RV-A dominance from May to July 2021 was accompanied by the replacement of A101 by A1B. RV-B6 constituted the main RV-B genotype during 2021. Remarkably, RV genotypes switched 15 months later, as most of the high-frequency genotypes from 2021 were not detected or were detected in a very low frequency in 2022.

Rarefaction analysis indicated that the genotype richness was well characterized during 2021, while in 2022 genotypic coverage lagged despite our sampling efforts (Supplementary Material, Supplementary Figure 2). Nonetheless, we covered >75% of estimated genotypic richness according to the coverage-based extrapolation curve. Based on the observed genotypic richness, Chao1 index calculation indicated the presence of up to 24 potentially unsampled genotypes in RV-A, 5 in RV-B, and 24 in RV-C (Supplementary Table 3).

### Novel RV-A and RV-C Genotypes Detected

We next examined the topology of VP1 trees for monophyletic clusters suggestive of novel genotypes (Supplementary Figure 3). Two monophyletic clusters exhibited distinctive patristic distances, and further analysis indicated that they had an average intergenotype *P* distance above the cutoff value of 0.13 [16]. We proposed them as genotypes A111 and C59. Genotype A111 exclusively comprised sequences from Washington State from 2017 to 2023, and C59 included sequences from Wisconsin from 2014 and Washington State from 2018 and 2023.

### Association of Clusters of Genotypes With Clinical and Demographic Characteristics

The high number of RV genotypes often complicates statistical association studies of clinical and demographic data. Therefore, we evaluated whether phylogenetically related groups of genotypes might demonstrate any such association. We performed principal component analysis based on a polyprotein-based pairwise genetic distance matrix applying unsupervised hierarchical clustering. Genotypes were grouped into 4 clusters in RV-A, 4 clusters in RV-B, and 7 clusters in RV-C (Supplementary Material, Supplementary Figure 4). While clusters showed no association with symptom, sex, or geographic location, they were associated with the age of the individuals. Specifically, for RV-A, clusters 1 and 2 exhibit an age group pattern different from cluster 3, which is more prevalent in the 5- to 18-year-old group. In contrast, cluster 4 is highly prevalent in children <5 years old (*G* test of independence, *P* < .05).

In RV-B, cluster 2 (genotype B6) is more associated with younger individuals as compared with the other 3 clusters (*G* test of independence, *P* < .05). This age association is further supported when age is analyzed as a continuous variable, with a median age of 11 years for cluster 2 as opposed to median ages of 31, 37, and 38 years for clusters 1, 3, and 4, respectively (Kruskal-Wallis test, *P* = .000001).

In RV-C, cluster 7 (C15) was more associated with individuals aged >65 years as compared with clusters 1 and 6. Despite their genetic similarity, these 3 clusters were more closely related to one another than to other genotypes (*G* test of independence, *P* < .05). We also evaluated the seasonality of the cluster of genotypes and found that RV-A and RV-B clusters were clearly distinguished by the year of sample collection (*P* < .05). Specifically, clusters 1 (A101) and 3 (A1B) in RV-A and clusters 2 (B6) and 4 (B27) in RV-B were associated with detections in 2021 when compared with other clusters. In the case of RV-C, the comparison by clusters did not result in a single supported association; however, cluster 2 was mainly detected in 2021, while clusters 6 and 7 were mainly detected in 2022.

### Recombination Between A105 and A21 Genotypes Within the 3C Protease Genetic Region

**Figure 5.**
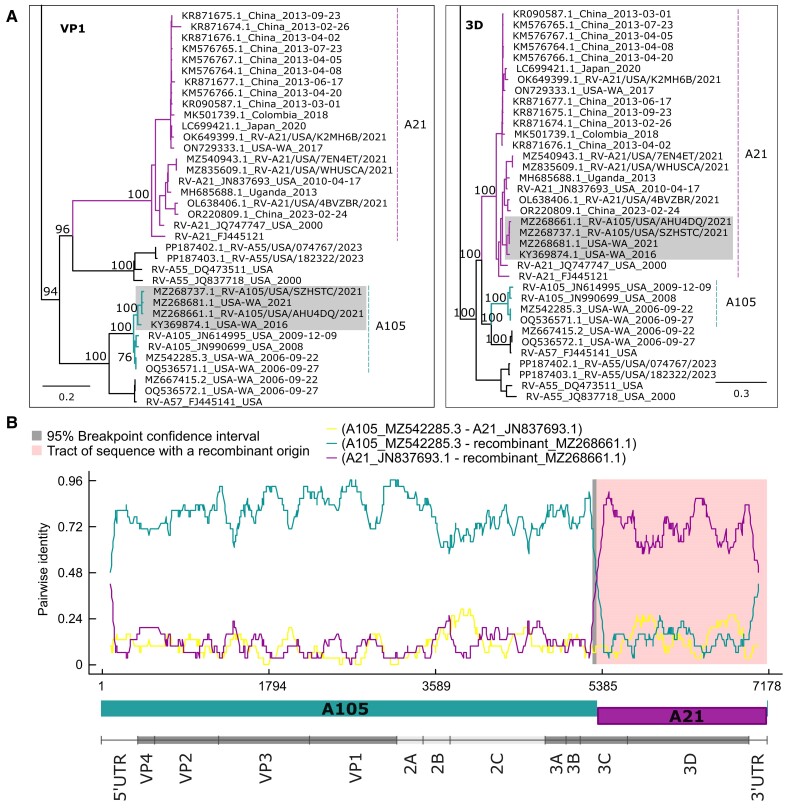
Recombination between RV-A105 and RV-A21 genotypes. *A*, Phylogenetic clades from the maximum likelihood trees with VP-1 and 3D nucleotide sequences. Branches highlight the inferred major (dark green) and minor (violet) parents for the recombinant clade (gray). Bootstrap values of relevant nodes are indicated in the nodes. Scale bar indicates substitution per site. *B*, pairwise sequence identity plot among the sequences MZ542285 (A105, major parent), JN837693 (A21, minor parent), and MZ268661 (recombinant). The gray background denotes the 95% recombination breakpoint confidence interval, and the pink background highlights the region with recombinant origin. The bottom of the plot represents the parent combination and the RV genome organization following the same scale of nucleotide positions. RV, rhinovirus.

We next analyzed the complete genomes of the RV circulating in Puget Sound to examine for noncapsid variation. Topologic comparison of VP1 and 3D/RdRp phylogenetic trees revealed inconsistencies in a monophyletic clade comprising 4 sequences from Washington State dating from 2016 and 2021. The clade was associated with the A105 VP1 capsid sequence and A21 3D polymerase sequence (Figure 5*A*). Further analysis identified nucleotide 5250 (95% CI, 5216–5271; GenBank MZ268661), corresponding to amino acids 37 to 55 of the 3C protease, as the likely recombination breakpoint, which was confirmed by all algorithms tested with Recombination Detection Program software (*P* < 10^−13^; Figure 5*B*).

### Intragenotypic RV Evolution and Variability

To unravel the history and diversity of intragenotype transmission, we reconstructed genome-based phylodynamic trees for the highly prevalent genotypes in this study. Genotypes A22, A29, A31, A46, B92, and C44 were not analyzed since they include <15 genomes with an available collection year. Genotypes A25, A39, and A67 showed no temporal signal.

The constructed phylodynamic trees showed similar evolutionary rates ranging from 1.3 × 10^−3^ to 1.1 × 10^−2^ substitutions per site per year (Supplementary Figure 5), coinciding with intergenotype evolutionary rates previously published [25, 26]. The topology of the trees indicates that the RVs in Puget Sound either were associated with distinct clades or formed a single clade that underwent significant divergence over time, as evidence by the date of the most recent common ancestor (MRCA). Comparison of MRCA dates for all Puget Sound sequences within each genotype revealed 2 distinct scenarios (Supplementary Figure 6). For certain genotypes, Puget Sound sequences were closely related, with MRCA dating up to a decade before sample collection date (“close common ancestor”). Conversely, for other genotypes, the MRCA of all Puget Sound sequences dated back several decades, suggesting that these RV strains belong to clades with a different evolutionary history. Therefore, although contemporary sequences within the same genotype share genetic similarity, they can derive from divergent intragenotypic clades dating back as far as 40 years ago.

We evaluated the amino acid variability to assess antigenic drift. Selection pressure estimation across the RV polyprotein for the high-frequency genotypes revealed an overall strong negative selection spanning the entire open reading frame for all genotypes. However, positively selected sites primarily within the C-terminal region of the VP1 capsid protein were identified in 6 genotypes. Specifically, positively selected VP1 residues were detected at positions 91 (genotype B92), 267 (genotype C1), 268 (genotype A29), 270 (genotype A31), 276 (genotype A30), and 282 (genotype A1B). Additionally, positively selected sites were found in the VP2 protein at residues 72 (genotype C1), 152 (genotype A31), and 157 (genotype B70) and VP3 at positions 60 (genotype C1) and 138 (genotype A30). Nonstructural proteins also exhibited positively selected sites, including P2-A at residues 25 (genotype C42), 30 (genotype A24), and 63 (genotype C3); the 3B protein at residue 6 (genotype C17); and the 3D protein at residues 67 (genotype C3), 274 (genotype B6), and 361 (genotype A31).

We further evaluated overall amino acid diversity within the Puget Sound data set, for which Shannon entropy was calculated with the sequences obtained in this study. There was moderate entropy in certain residues within the VP1 protein, including residue 91 in genotype B92, residue 130 in genotype A31, and residue 87 in genotype C15, as well as residue 250 in the 3C protein of genotype A31, as indicated by Shannon entropy values exceeding 1 (Supplementary Figure 7). Notably, all residues in other genotypes consistently exhibited entropy values <1, suggesting a highly conserved polyprotein sequence across all genotypes.

## DISCUSSION

In this study, we analyzed the largest collection of newly sequenced RV genomes to date, comprising more than half of publicly available RV genomes in NCBI GenBank as of December 2023. We observed a high RV incidence during the studied period, even during November and December 2022 when the “tripledemic” of SARS-CoV-2, respiratory syncytial virus, and influenza virus caused significant public health concern [27].

Our study supports previous findings indicating that symptomatology is associated with higher viral loads than asymptomatic status and particularly in older adults [28–30]. We observed a high incidence of RV-A in spring and summer, with RV-C predominating during the winter, consistent with prior reports [31]. However, unlike previous reports, we did not find age groups or symptomatology to be associated with specific RV species [3]. Our findings were limited to mild outpatient cases taken from community testing sites, and broader surveillance in severe infections could yield different results. Our study showed that the containment measures against the COVID-19 pandemic had no effect on the geographically spread of RV species, cocirculation, and replacement in the Puget Sound region, consistent with the high positivity rate seen for RV in hospital-based surveillance data. Further investigations globally are needed to assess if the seasonality that we observed reflects a local or global circulation pattern, helping to determine if RV spread extends beyond strict border containment measures.

Across 2021 to 2022, we detected 99 of 173 described RV genotypes (Supplementary Figure 8). However, the distribution of these genotypes was highly skewed, with 16 genotypes comprising >85% of RV-A cases, 4 genotypes comprising >75% RV-B, and 9 genotypes comprising >75% of RV-C. Despite our sequencing efforts, >70 historically described genotypes and approximately one-quarter of RV genotypes based on rarefaction analysis remain undetected. These findings underscore the challenges in designing effective molecular surveillance for RV.

Our genomic analysis detected an A105-A21 recombinant RV with a breakpoint in the 3C protein region. A similar 3C recombination breakpoint has been reported between A76 and A56 genotypes [25, 26]. This region of the genome is a hot spot for recombination events in poliovirus [32]. However, our A105-A21 recombination breakpoint was located upstream of the corresponding RNAse L inhibitory RNA secondary structure element, which was shown to be associated with higher rates of recombination in poliovirus [33, 34]. Further investigation of the RNA secondary structure of RV may help to explain our findings.

Overall, genotypes that predominated in 2021 were replaced in 2022. However, we did not perform serologic analysis to determine whether population-level immunity to prevalent genotypes in 2021 defined the genotypic incidence the following year or if it is related to another seasonality pattern. Prior work has shown that RV serotype–specific IgA in serum can last for at least 1 year, indicating that immunity is likely a contributing factor for shifting genotypic incidence [35].

Within RV genotypes, viruses phylogenetically related into different clades were detected, with cocirculation of RVs from the same genotype that have diverged up to 40 years ago. However, the polyprotein sequence exhibited a significant constraint on amino acid variability. A limited number of positively selected residues in the viral proteins of 12 high-frequency genotypes were found, mainly in the C-terminal region of VP1. The VP1 protein is the major architectural component of the viral capsid and host-cell receptor attachment, suggesting that it can be under some degree of antigenic drift. Further studies are needed to evaluate the biological impact of amino acid changes in these sites, but overall results suggest that the intragenotypic amino acid variability is low, a promising fact for the development of an RV vaccine.

Our study emphasizes the importance of investigating correlations between genotypes and serotypes to understand long-term immunity and cross-protection. Recent research by Bochkov et al revealed cross-protection among some genetically similar genotypes in RV-A and RV-C species [36]. This fact was originally mentioned in 1982 when Cooney et al demonstrated cross-protection among 50 RV types, categorizing them into 16 antigenic groups [37]. In our study, hierarchical clustering based on polyprotein genetic distances helped to associate some genotypes into a small number of clusters. We found a statistical association of some clusters with the year of detection, which might indicate a relationship with long-term immunity interference. While this clustering does not necessarily define antigenic and serotypic relatedness, it did cluster RV-A12, RV-A20, and RV-A78, which have shown cross-neutralizing protection [36].

This study has several limitations: convenience sampling of remnant SARS-CoV-2–negative specimens, restriction of sampling to outpatient community testing sites, limited clinical metadata associated with high-throughput drive-through testing, limited sampling during the 2021–2022 period within the Puget Sound area, and testing of 4-sample pooling in 2021 where only half of the individual RV-positive specimens were identified. Nonetheless, the high volume of outpatient respiratory testing performed during the SARS-CoV-2 pandemic and the omnipresent high cocirculation of RV made this study possible.

Overall, our deep look at RV genomic epidemiology during the 2021–2022 period in Puget Sound significantly increased the availability of genomic data for RV. We highlighted the seemingly unrestricted ability of RV genotypes to cocirculate despite public health containment measures that significantly limited cocirculation of other respiratory viruses. In addition, we showed the importance of updating RV surveillance to a genomic approach to monitor recombination, as well as the continued need to examine the antigenic and serotypic relatedness of RV genotypes that may reveal relationships with viral seasonality and vaccine development.

## Supplementary Data

Supplementary materials are available at *The Journal of Infectious Diseases* online (http://jid.oxfordjournals.org/). Supplementary materials consist of data provided by the author that are published to benefit the reader. The posted materials are not copyedited. The contents of all supplementary data are the sole responsibility of the authors. Questions or messages regarding errors should be addressed to the author.

## Supplementary Material

jiae347_Supplementary_Data

## Notes

***Acknowledgments.*** We thank the excellent technical support of Nathan Breit and Carlos Avendaño. This research received no specific funding and was supported by departmental funds.

***Financial support*.** No financial support was received for this work.
