# Supplementary material for: Genomic Epidemiology and Evolution of Rhinovirus in Western Washington State, 2021–2022
Source: J Infect Dis. 2024 Jul 4;231(1):e154–64. doi: 10.1093/infdis/jiae347 (PMC11793040; doi:10.1093/infdis/jiae347)
Supplement: jiae347_Supplementary_Data [file jiae347_supplementary_data.zip › RV_SupplementaryMaterial_R1_20240621_bibliography.docx]

**Genomic epidemiology and evolution of rhinovirus in western Washington State, 2021-22**

**Supplementary Material**

**Supplementary Methods**

*Clinical samples and data collection*

We retrospectively examined remnant SARS-CoV-2-negative nasal swabs collected from outpatients attending University of Washington (UW) Medicine COVID-19 community testing sites. RT-qPCR screening was performed in two periods. From February to July 2021, we screened 33,528 samples organized into 8,328 4-sample pools. From these, 3,023 pools were RV-positive. Of these, 1,296 positive pools were further analyzed to determine the positive samples individually. We obtained 1,044 pools with one positive sample, 233 with two, 18 with three and 1 with four, resulting a total of 1,568 individually confirmed RV-positive samples. Samples pool with a Ct value below 33 underwent next-generation sequencing (NGS). Genomic analyses were performed using confirmed consensus genomes from single positive samples within each pool. During November and December 2022, we individually screened 10,656 randomly selected samples and sequenced specimens with Ct value < 33. Additionally, twelve samples with Ct values between 33 and 37 were also sequenced.

Demographic and symptomatic information were collected from optional COVID-19 testing screening questionnaires. A symptomatic respiratory infection was considered when the individual declared at least one respiratory symptom at the time of sample collection. Individuals declaring no respiratory symptoms but a close contact with a COVID-19 positive individual or requesting negative SARS-CoV-2 confirmation before a trip were identified as asymptomatic. Less commonly reported symptoms (<5% of cases) included sneezing, body ache, fatigue, difficulty breathing, nausea and vomiting.

*Rhinovirus RT-qPCR*

Viral RNA was extracted from nasal swabs using the MagNA Pure 96 System (Roche). Extracted RNA underwent to RT-qPCR for RV with AgPath-ID One-Step RT-PCR (Life Technologies) or Luna Universal Probe One-Step RT-qPCR Kit (New England Biolabs) according to manufacturer recommendation, after demonstrating high correlation of same-sample RV Ct values between two kits. Primers and probes targeting the 5´ UTR of the RV genome were previously published[1,2]:

Primer -Forward: 5´-CP[A] GCC [T]GC GTG GY-3´

Primer -Reverse: 5´-GAA ACA CGG ACA CCC AAA GTA-3´

Probe: 5´-TCC TCC GGC CCC TGA ATG YGG C-3´

P= pyrimidine derivative, [A]= LNA-dA (Locked Nucleic Acid, LNA), [T]= LNA-dT, and Y= C/T

After sequencing RV genomes, we examined sequences for any mutations in the region where the primers and probe annealed to check for changes in Ct values/viral load quantitation. We detected the following mismatches in the forward primer (the mismatch is highlighted in underlined bold):

**G**PAGCCTGCGTGGY: RV-C19, RV-C55, RV-C35, RV-C20, RV-C26, RV-C34, RV-C54, RV-C59

**G**PAGCC**C**GCGTGGY: RV-C33, RV-C36, RV-C46, RV-C9, RV-C53

CP**T**GCCTGCGTGGY: RV-C11, RV-C15, RV-C23, RV-C25, RV-C2, RV-C40

CPAGCC**C**GCGTGGY: RV-A101, RV-C21, RV-C28, RV-C18, RV-C42, RV-C56, RV-C7, RV-C8, RV-C31

**T**PAGCCTGCGTGGY: RV-B42, RV-B48, RV-B70, RV-B91, RV-C43

We detected the following mismatches in the reverse primer (the mismatch is highlighted in bold):

GAAACACGG**TT**ACCCAAAGTA: RV-C20, RV-C34

GAAACACGGACA**T**CCAAAGTA: RV-A59

We detected the following mismatches in probe (the mismatch is highlighted in bold):

TCCTCCGGC**T**CCTGAATGYGGC: RV-A59

TCCTCCGGCCCCTGAATGYGGC: RV-A9, RV-A63

TCCTCCGG**T**CCCTGAATGYGGC: RV-C20, RV-C34

To evaluate whether the mismatches regarding the sequences of primers and probe affected the sensitivity of the RT-qPCR assay we compared the Ct values of the genotypes with and without mismatches. Results are available at <https://github.com/greninger-lab/HRV_epidemiology>. The average Ct for each genotype was variable, but there were no pattern associating high Ct values with the genotypes carrying mutations in the primers or probe, indicating the Ct variability would be related with another factor.

*NGS and assembly of viral consensus genomes*

RNA from nasal swabs with RV Ct value below 33 were sequenced by metagenomic next-generation sequencing as described previously. Twelve samples with Ct values 33-37 were also sequenced. Briefly, viral RNA was extracted with Quick-RNA Viral Kit (Zymo Research), double stranded cDNA synthesis was performed with random hexamers with SuperScript IV Reverse Transcriptase and Sequenase Version 2.0 DNA Polymerase (ThermoFisher Scientific) and purified with AMPure XP Magnetic Beads (Beckman Coulter). Library preparation was performed with Illumina DNA Prep, (M) Tagmentation kit (Illumina) and sequenced as 2×100-bp or 2×150-bp runs on a NovaSeq sequencer (Illumina).

Viral consensus genomes were called using the custom pipelines <https://github.com/Paul-rk-cruz/HRV_Pipeline> and <https://github.com/greninger-lab/revica> to map NGS reads against a viral reference database of all existing RV genomes from the International Committee on Taxonomy of Viruses (ICTV) as of March 2022. First, mapping against the viral reference database was performed, followed by one round of mapping against the viral sequence with the highest median coverage and two iterations of mapping against the consensus reconstruction. Consensus genomes were generated if the alignment covered above 60% of the reference with a minimum depth of 3X and minimum base quality of 20 (Supplementary Table 1).

*Sequence analysis*

RV complete genomes longer than 6,000 nt from human hosts were downloaded from NCBI Virus (May 25, 2023) using the taxonomic IDs 147711 (RV-A), 147712 (RV-B), 463676 (RV-C). Partial genomes were downloaded from the *Picornaviridae* Study Group database to represent genotypes without complete genome available (https://www.picornastudygroup.com/). MEGA11 software was used to calculate the pairwise genetic p-distance for RV genotypes with 1,000 replicates bootstrapping [3]. Alignment-based recombination analysis was performed with RDP4 software using the RDP, GENECONV, Bootscan, Maximum Chi Square, Chimaera, SiScan, and 3SEQ methods with default settings [4]. Comparative co-phylogeny of VP1 and 3D regions was performed using ‘cophylo’ tool from the ‘phytools’ package in R [5].

*Statistical analyses*

Rarefaction curves and the coverage-based extrapolation curves were calculated to estimate the genotypic diversity using the packages ‘vegan’ and ‘iNEXT’ in RStudio 2022.07.2 [6,7]. Association among categorical and continuous variables were evaluated with ANOVA, Kruskal-Wallis test, Wilcoxon test, G-test, Chi-squared test, or Wald odds ratio using AMR or epitools libraries in RStudio [8]. Principal component analysis (PCA) and hierarchical clustering (HC) based on pairwise genetic distances (p-distance) were performed using the packages ‘FactoMineR’, ‘factoextra’ and ‘eclust’ in RStudio (Supplementary Material). R markdown code is available on GitHub (https://github.com/greninger-lab/HRV_epidemiology).

*Phylogenetic analyses*

Sequences were aligned with MAFFT v7.490 and visualized with Aliview v1.28 to detect and correct alignment artifacts, mainly around nucleotide gaps regions [9,10]. Maximum likelihood trees were inferred with IQ-TREE v2.1 using SH-aLRT test (1,000 replicates) and UFBoot2 method (1,000 replicates) to evaluate reliability of phylogenetic clades [11,12]. Monophyletic clusters (clades) were considered as statistically supported when UFBoot2 value ≥ 90% and SH-aLRT ≥ 80%. Phylogenetic tree visualization was performed with Figtree v1.4.4 (<https://github.com/rambaut/figtree>).

The evolutionary rate was inferred with BEAST2 package v2.7.5 using an strict or optimized relaxed clock defined with TempEst v1.5.3 [13], the substitution model was selected with ModelFinder and the tree priors selected according to the Nested Sampling test [14,15]. The convergence of the BEAST inference was assessed from the estimations of the Effective Sampling Size (ESS) and the highest posterior density interval (95% HPD) after a 10% burn-in using Tracer v1.7. When required, LogCombiner was used to combine up to four runs, each of them with 50 million generations of Markov chain Monte Carlo (MCMC) with a sample frequency to obtain 10,000 sampled trees. TreeAnnotator was used to summarize the information from the sampled trees onto a single tree (the maximum clade credibility tree, MCCT). Tree clades from BEAST were considered statistically supported when the posterior probability was ≥0.8.

*Evaluation of RV genotype classification with neighbor joining and maximum likelihood inferences*

We used the VP1 reference sequences reported by ICTV and the Picornaviridae Study Group database (<https://www.picornastudygroup.com/>) to evaluate the reliability of genotyping despite the inference methodology used. Specifically, we compared the tree topology obtained with neighbor joining (NJ) and maximum likelihood.

NJ trees were constructed with MEGA11 (Molecular Evolutionary Genetics Analysis) software using p-distance method, uniform rates among sites and pairwise deletion of gaps/missing data [3]. We included 1,000 Bootstrap replicates to assess the reliability of phylogenetic clades.

Maximum likelihood trees were constructed with IQ-TREE v2.1, using ModelFinder to select the suitable nucleotide substitution model and SH-aLRT test (1,000 replicates) and UFBoot2 method (1,000 replicates) to evaluate reliability of phylogenetic clades [11,12,15]. Phylogenetic clades were considered as statistically supported when UFBoot2 value ≥ 90% and SH-aLRT ≥ 80%.

A total of 197 RV-A, 67 RV-B and 81 RV-C sequences were used for this analysis. The constructed trees are available at https://github.com/greninger-lab/HRV_epidemiology and the sequences names contain the NCBI GenBank accession number.

The comparison was evaluated with tanglegram plots (representation of the co-phylogeny in which the two phylogenetic trees are linked by the tips) constructed in RStudio with the package ‘phytools’ [5]. The trees were visually inspected with FigTree v1.4.4 (<https://github.com/rambaut/figtree>). Constructed trees and tanglegrams are available at <https://github.com/greninger-lab/HRV_epidemiology>.The clades association was consistent between different inferences for the three RV species, with minimal topological differences and a robust statistical support for genotype-defining nodes. Considering the results, we used IQ-TREE software (maximum likelihood inference) throughout our study since its suitability to deal with large datasets [11].

*Rarefaction and extrapolation curves to evaluate the RV genotype diversity (richness) covered in this study*

The coverage of RV genotypic diversity per year and month of sample collection was estimated with rarefaction curves with the packages ‘vegan’ and the coverage-based extrapolation curves with the package ‘iNEXT’ in RStudio [6,7]. Details on the code used is available in the R Markdown files RV_rarefaction.Rmd and RV_rarefaction.html at https://github.com/greninger-lab/HRV_epidemiology, including the data frames.

Rarefaction curves were calculated with a step size of 1. Supplementary Figure 2 shows the results of the RV genotypes per year of sample collection, and the curves of the genotypes per species and month of sample collection. In 2021 the genotype richness was well characterized denoted by a curve reaching the plateau, while during 2022 the curve still increased meaning that our sequencing depth was not enough, and more genotypes may be detected with more sequencing efforts (Supplementary Figure 2). The evaluation per month and RV species detailed in more depth the situation, marked by the rarefaction curve proximate to the asymptote in some but not all RV species for a given month. For example, while in June 2021 the sampling size optimally describes the genotypic diversity of RV-A, in RV-B and RV-C species the genotypic richness is not totally represented.

In addition, estimation of genotype richness (Hill number q=0) was performed on the individual-based abundance data. Then the coverage-based extrapolation curve was performed. Bottom of Supplementary Figure 2 shows the genotype richness estimator per year and the estimation per RV species and month of sample collection, as a function of sample coverage. Solid lines of the curves represent rarefaction while dashed curves represent extrapolation beyond observed samples. The shaded areas highlight the 95% confidence intervals calculated from bootstrap. The results show that most of the times our sampling efforts covered more than 75% of estimated genotypic diversity (Supplementary Figure 2). We estimated the total number of genotypes per species for the months covering more than 75% diversity using the Chao1 index inference in ‘iNEXT’ package. The calculation indicated a range of 18-44 genotypes monthly co-circulating in RV-A, 2-12 genotypes in RV-B and 15-41 genotypes in RV-C (Supplementary table 3).

**Characterization of the new genotypes A111 and C59.**

During the phylogenetic analysis interpretation, three distinctive monophyletic clusters were observed with no close genotype reference sequence. The patristic distances (branch length) of the clades were equivalent to those differentiating genotypes, suggesting potential novel genotypes. Further analysis with recently new reported genotypes, but not confirmed by ICTV, identified an RV-B sequence from November 2022 as B107 genotype. Nevertheless, the other two clades did not have a close reference genotype. In Supplementary Figure 3, partial phylogenetic trees of RV-A (8A) and RV-C (8B) are shown with details about the supported clades in the nodes. Gray highlights the closest genotype/s including the genotype’s name at the left. Red boxes highlight the clade of the new genotypes, including the genotype’s name at the right. Scale bar indicates substitution per site.

At the right of each subtree, the evolutionary divergence, calculated as average VP1 p-distance between and within genotypes is informed in bold and the standard error estimation in blue and italics. Average VP1 p-distance between clades above 0.13 (<87% identity) indicates they are new genotypes.

**Principal component analysis (PCA) and hierarchical clustering**

We evaluated whether a group of genotypes instead of independently were correlated using principal component analysis (PCA) based on the polyprotein-based pairwise genetic distance (p-distance) matrix as a categorization complexity between species and genotypes. The pairwise genetic distance (p-distance) matrix was calculated for each RV species with the package ‘ape’ in RStudio based on a nucleotide alignment of the polyprotein gene. PCA was calculated and evaluated with the package ‘FactoMineR’ and ‘factoextra’ in RStudio. Details about the pipeline used are available at https://github.com/greninger-lab/HRV_epidemiology. We analyze with hierarchical clustering (HC) whether unsupervised clustering (package ‘eclust’ in RStudio) of the data can be explained with the clinical and demographic characteristics of the individuals: age group, sex, symptoms presence and geographic location of the COVID-19 community testing sites. HC was calculated based on complete-linkage method and Euclidean distance, assessing the number of clusters with WSS (within sum of squares) and Silhouette score methods. HC grouped the sequences into 4 clusters in RV-A, 4 clusters in RV-B and 7 clusters in RV-C (Supplementary Figure 4, numbered clusters identified with colors and below each cluster the group of genotypes is detailed). The HC clustering showed no relation with the symptom presence, individual’s sex, or geographic location (detailed results available at <https://github.com/greninger-lab/HRV_epidemiology>). However, supported correlation of the genotype clusters with the age and the year of sample collection was found.

**Analysis of the intra-genotypic RV variability.**

Selection pressure within the RV polyprotein was calculated for each high frequency genotype independently. The datasets were nucleotide alignments of genomes from 2021 and 2022 collected in Washington State together with all the genomes from the same genotype available in public databases. The method used for the calculation was FUBAR to infer nonsynoymous (dN) and synonymous (dS) substitution rates on a per-site basis for the polyprotein coding alignment and the corresponding phylogeny within the online website <https://www.datamonkey.org/> [16]. Result of the selection pressure estimation per position and genotype are available at https://github.com/greninger-lab/HRV_epidemiology.

Shannon entropy was calculated online at <https://www.hiv.lanl.gov/> for each prevalent genotype using the Puget Sound sequences alignment of the open reading frame of the RV polyprotein translated with the standard genetic code. Supplementary Figure 7 shows the Shannon entropy value as bars at each position of the entire polyprotein. Values above 1 were considered moderate entropy and below 1 were considered conserved positions.

Location of the amino acids into the mature proteins listed in the main text referred to each genotype references with NCBI GenBank accession numbers D00239 (A1B), FJ445190 (A24), FJ445125 (A29), FJ445179 (A30), FJ445126 (A31), JX193795 (B6), DQ473489 (B70), FJ445169 (B92), EF186077 (C1 and C3), JN815240 (C3), GU219984 (C15), JN815240 (C17) and JQ994600 (C42).

**Bibliography**

1. Weinberg GA, Schnabel KC, Erdman DD, et al. Field evaluation of TaqMan Array Card (TAC) for the simultaneous detection of multiple respiratory viruses in children with acute respiratory infection. J Clin Virol. **2013**; 57(3):254–260.

2. Sedlak RH, Nguyen T, Palileo I, Jerome KR, Kuypers J. Superiority of Digital Reverse Transcription-PCR (RT-PCR) over Real-Time RT-PCR for Quantitation of Highly Divergent Human Rhinoviruses. J Clin Microbiol. **2017**; 55(2):442–449.

3. Stecher G, Tamura K, Kumar S. Molecular Evolutionary Genetics Analysis (MEGA) for macOS. Molecular Biology and Evolution. **2020**; 37(4):1237–1239.

4. Martin DP, Murrell B, Golden M, Khoosal A, Muhire B. RDP4: Detection and analysis of recombination patterns in virus genomes. Virus Evol. **2015**; 1(1):vev003.

5. Revell LJ. phytools: an R package for phylogenetic comparative biology (and other things). Methods in Ecology and Evolution. **2012**; 3(2):217–223.

6. Oksanen J, Simpson GL, Blanchet FG, et al. vegan: Community Ecology Package version 2.6-4 from CRAN. 2022. Available from: https://rdrr.io/cran/vegan/

7. Chao A, Gotelli NJ, Hsieh TC, et al. Rarefaction and extrapolation with Hill numbers: a framework for sampling and estimation in species diversity studies. Ecological Monographs. **2014**; 84(1):45–67.

8. Sullivan KM, Dean A, Soe MM. OpenEpi: A Web-based Epidemiologic and Statistical Calculator for Public Health. Public Health Rep. **2009**; 124(3):471–474.

9. Katoh K, Standley DM. MAFFT Multiple Sequence Alignment Software Version 7: Improvements in Performance and Usability. Mol Biol Evol. **2013**; 30(4):772–780.

10. Larsson A. AliView: a fast and lightweight alignment viewer and editor for large datasets. Bioinformatics. **2014**; 30(22):3276–3278.

11. Minh BQ, Schmidt HA, Chernomor O, et al. IQ-TREE 2: New Models and Efficient Methods for Phylogenetic Inference in the Genomic Era. Molecular Biology and Evolution. **2020**; 37(5).

12. Hoang DT, Chernomor O, Haeseler A von, Minh BQ, Vinh LS. UFBoot2: Improving the Ultrafast Bootstrap Approximation. Molecular Biology and Evolution. **2018**; 35(2):518–522.

13. Rambaut A, Lam TT, Max Carvalho L, Pybus OG. Exploring the temporal structure of heterochronous sequences using TempEst (formerly Path-O-Gen). Virus Evolution. **2016**; 2(1):vew007.

14. Bouckaert R, Vaughan TG, Barido-Sottani J, et al. BEAST 2.5: An advanced software platform for Bayesian evolutionary analysis. PLOS Computational Biology. Public Library of Science; **2019**; 15(4):e1006650.

15. Kalyaanamoorthy S, Minh BQ, Wong TKF, Haeseler A von, Jermiin LS. ModelFinder: fast model selection for accurate phylogenetic estimates. Nat Methods. Nature Publishing Group; **2017**; 14(6):587–589.

16. Murrell B, Moola S, Mabona A, et al. FUBAR: A Fast, Unconstrained Bayesian AppRoximation for Inferring Selection. Molecular Biology and Evolution. **2013**; 30(5):1196–1205.
