# Supplementary material for: Genomic Epidemiology and Evolution of Rhinovirus in Western Washington State, 2021–2022
Source: J Infect Dis. 2024 Jul 4;231(1):e154–64. doi: 10.1093/infdis/jiae347 (PMC11793040; doi:10.1093/infdis/jiae347)

Supplementary Figure 2. Rarefaction curves of the diversity of RV genotypes detected.

The rarefaction curve plots represent the number of genomes sampled against the genotypic richness detected. The coverage-based rarefaction (solid lines) and extrapolation (dashed lines) curve plots are based on species richness (Hill's numbers q=0) and include the 95% confidence intervals as shaded areas based on 200 replication bootstrap method. For both analysis, rarefaction and coverage-based extrapolation, all RV species per year of sample collection and each RV species per month of sample collection were analyzed.

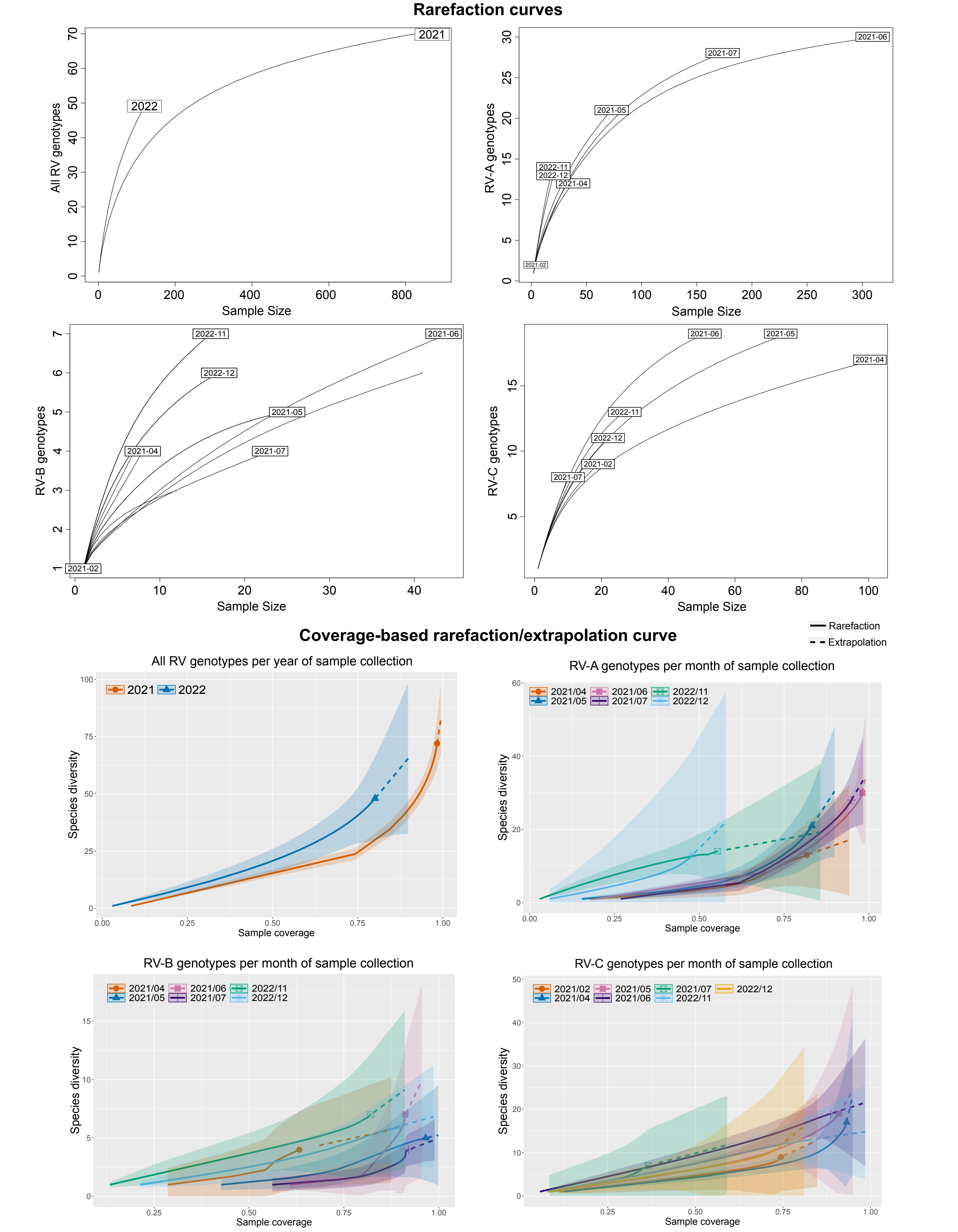

Supplement: jiae347_Supplementary_Data [file jiae347_supplementary_data.zip › SupplementaryFigure2_R1_202405.pdf]
