# Supplementary material for: Genomic Epidemiology and Evolution of Rhinovirus in Western Washington State, 2021–2022
Source: J Infect Dis. 2024 Jul 4;231(1):e154–64. doi: 10.1093/infdis/jiae347 (PMC11793040; doi:10.1093/infdis/jiae347)
Supplement: jiae347_Supplementary_Data [file jiae347_supplementary_data.zip › SupplementaryFigure3_R1_202405.pdf]

Supplementary Figure 3. Detection of the new genotypes A111 and C59.

Characterization of the new genotypes A111 (A) and C59 (B). Partial phylogenetic trees show the monophyletic clade statistically supported by ultrafast bootstrap (with value above 90) indicated with a red square and the closest genotype/s highlighted in gray. Scale bar indicates substitution per site. At the right of each subtree, the evolutionary divergence (calculated as average p-distance) between and within genotype clades is informed in bold and the standard error estimation in blue italics.

A

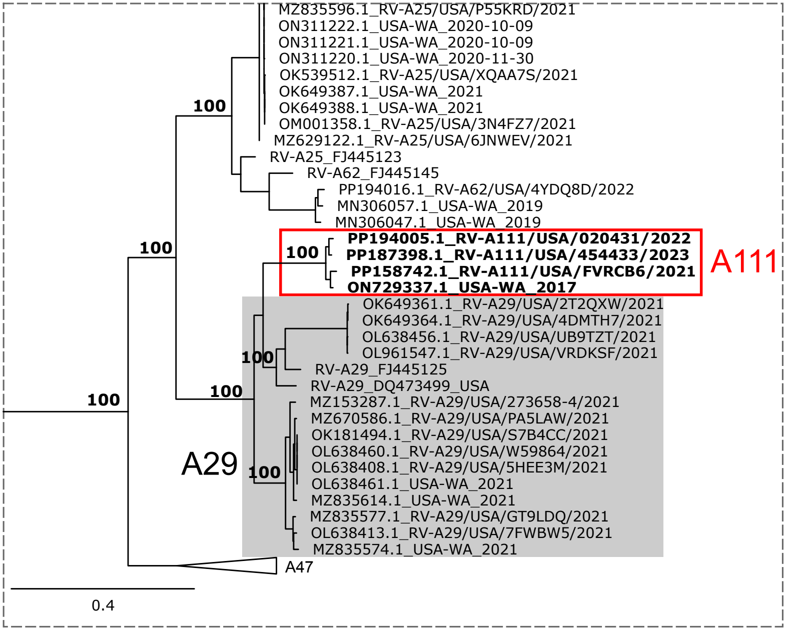

Average p-distance between clades

	A111	A29
A111		*0.009*
A29	**0.153**	

Average p-distance within clade

A111	**0.025**	*0.004*
A29	**0.087**	*0.006*

B

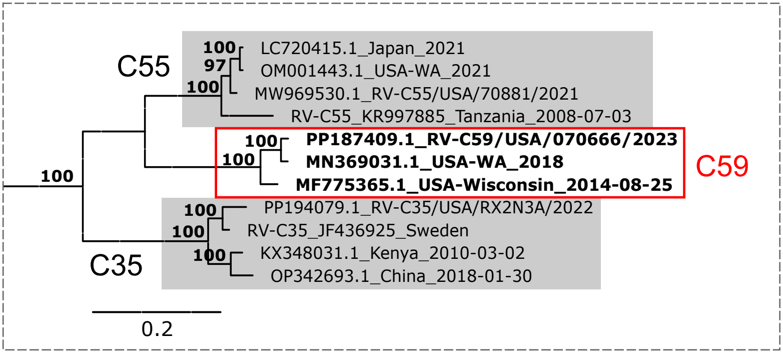

Average p-distance between clades

	C59	C35	C55
C59		*0.013*	*0.012*
C35	**0.224**		*0.013*
C55	**0.197**	**0.223**	

Average p-distance within clade

C59	**0.052**	*0.007*
C35	**0.078**	*0.007*
C55	**0.057**	*0.007*
