# Supplementary material for: Genomic Epidemiology and Evolution of Rhinovirus in Western Washington State, 2021–2022
Source: J Infect Dis. 2024 Jul 4;231(1):e154–64. doi: 10.1093/infdis/jiae347 (PMC11793040; doi:10.1093/infdis/jiae347)

Supplementary Figure 4. Hierarchical clustering analysis of the polyprotein-based pairwise genetic distance matrix. Principal component analysis (PCA) and hierarchical clustering was calculated from the pairwise genetic distance based on the polyprotein sequence for each RV species. The number of clusters was defined by within-cluster sum of squares method. Each numbered cluster is highlighted with a colored squared including the detail of the genotypes clustering together. At the right of each dendrogram the analysis of association between age of the individuals and the clusters obtained is shown.

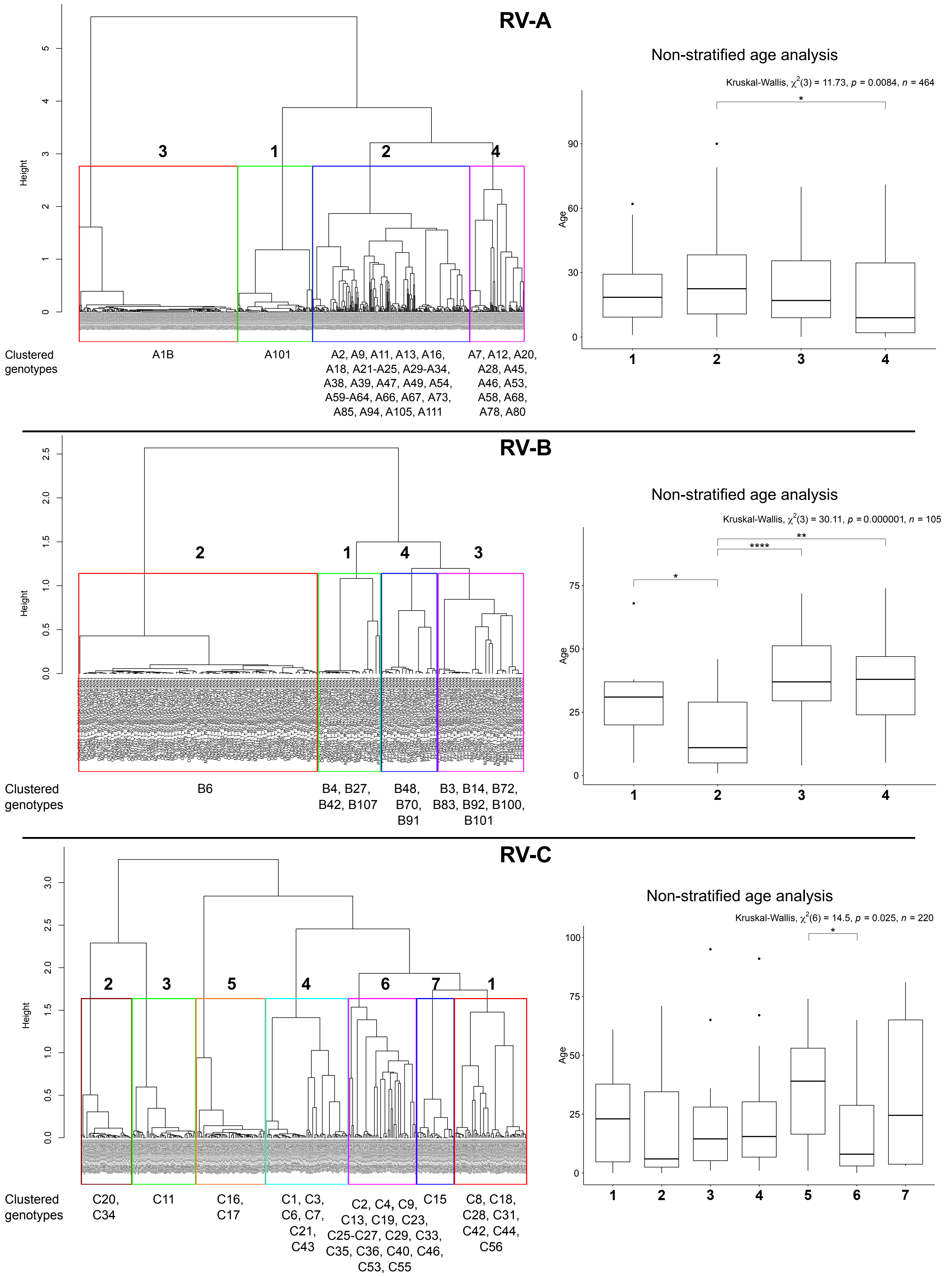

Supplement: jiae347_Supplementary_Data [file jiae347_supplementary_data.zip › SupplementaryFigure4_R1_202405.pdf]
