# Supplementary material for: Genomic Epidemiology and Evolution of Rhinovirus in Western Washington State, 2021–2022
Source: J Infect Dis. 2024 Jul 4;231(1):e154–64. doi: 10.1093/infdis/jiae347 (PMC11793040; doi:10.1093/infdis/jiae347)
Supplement: jiae347_Supplementary_Data [file jiae347_supplementary_data.zip › SupplementaryFigure5_R1_202406.pdf]

Supplementary Figure 5. Phylodynamic analysis of prevalent RV genotypes.

Maximum clade credibility tree (MCCT) with complete genomes of prevalent RV genotypes. The scale bar indicate the time in years and for some nodes the time of the most recent common ancestors (tMRCA) is detailed. The tMRCA uncertainty (95% highest posterior density intervals or 95% HPD) is indicated with blue bars. Genomes generated in this study are highlithed in red. The evolutionary rate with the 95%HDP is indicated below the name of each genotype. Clades comprising Washington State (WA) genomes only are collapsed into triangle including the number of collapsed sequences at the right.

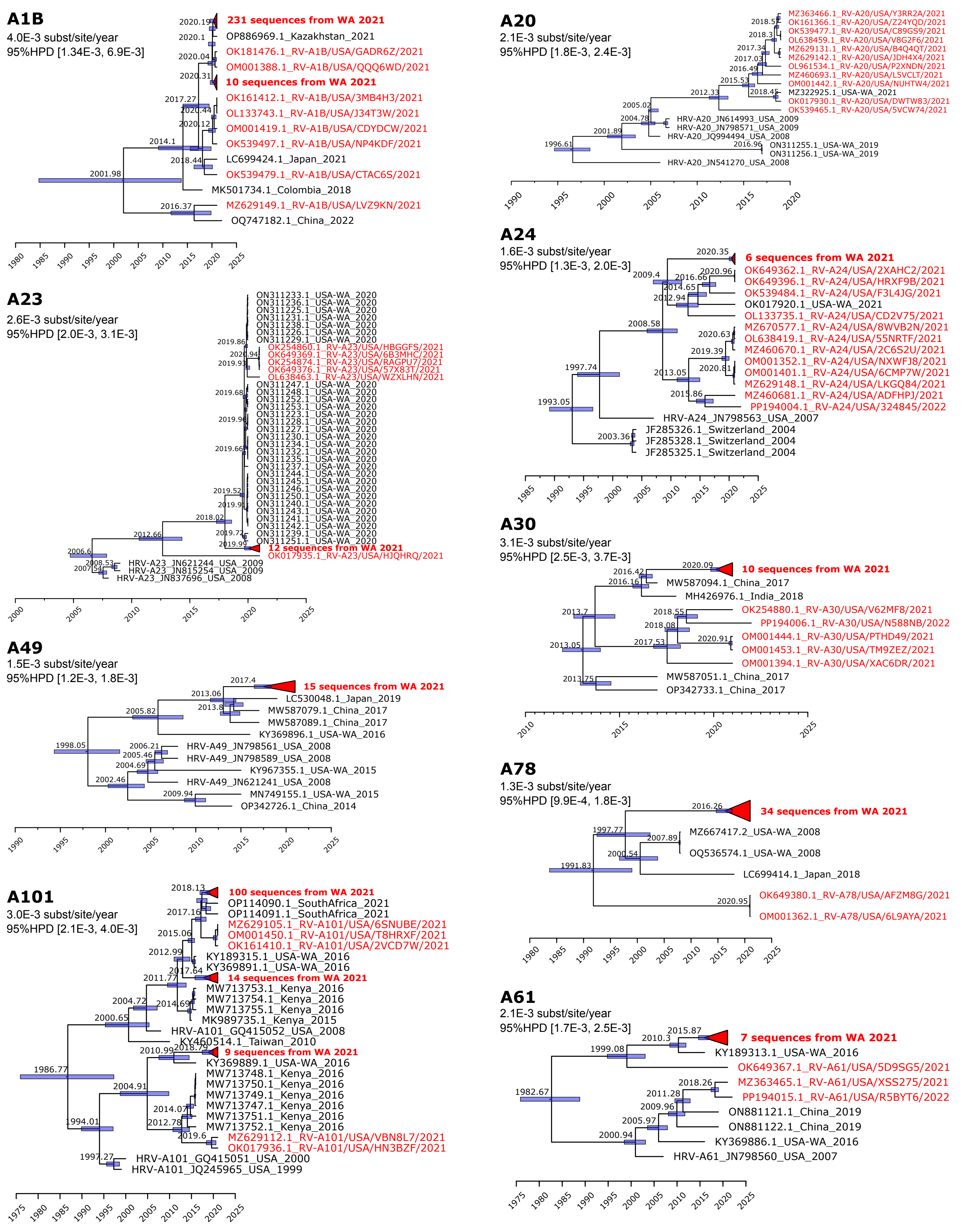

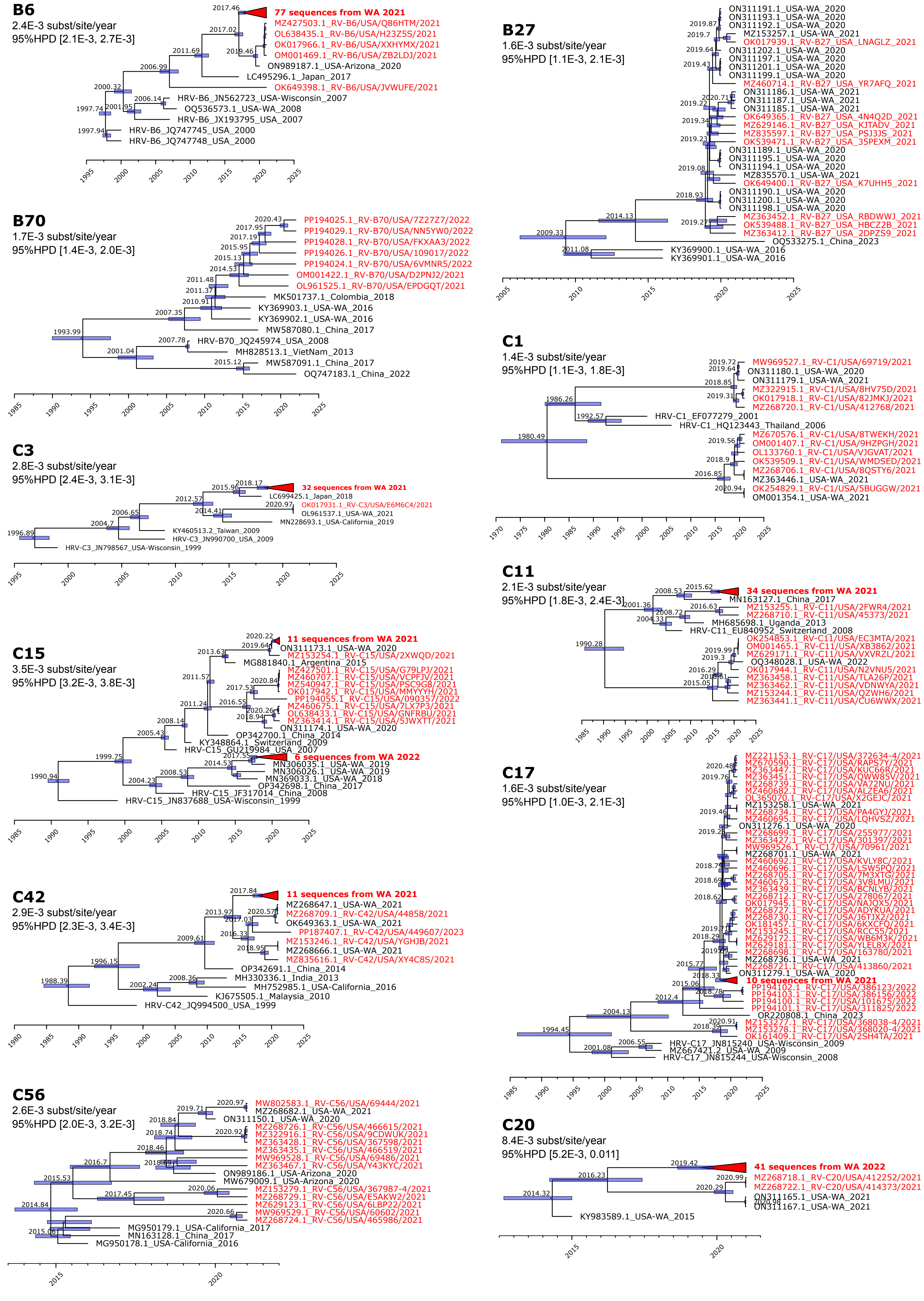

B6
2.4E-3 subst/site/year
95%HPD [2.1E-3, 2.7E-3]
77 sequences from WA 2021
MZ427503.1_RV-B6/USA/Q86HTM/2021
OL638435.1_RV-B6/USA/H23Z5S/2021
OK017966.1_RV-B6/USA/XXHYMX/2021
OM001469.1_RV-B6/USA/ZB2LDJ/2021
ON989187.1_USA-Arizona_2020
LC495296.1_Japan_2017
OK649398.1_RV-B6/USA/JVWUFE/2021
HRV-B6_JN562723_USA-Wisconsin_2007
OQ536573.1_USA-WA_2008
HRV-B6_JX193795_USA_2007
HRV-B6_JQ747745_USA_2000
HRV-B6_JQ747748_USA_2000
2017.46
2017.02
2019.46
2011.69
2006.99
2000.32
2006.14
1997.74
2001.95
1997.94
1995
2000
2005
2010
2015
2020
2025
B27
1.6E-3 subst/site/year
95%HPD [1.1E-3, 2.1E-3]
ON311191.1_USA-WA_2020
ON311193.1_USA-WA_2020
ON311192.1_USA-WA_2020
MZ153257.1_USA-WA_2021
OK017939.1_RV-B27_USA_LNAGLZ_2021
ON311202.1_USA-WA_2020
ON311197.1_USA-WA_2020
ON311201.1_USA-WA_2020
ON311199.1_USA-WA_2020
MZ460714.1_RV-B27_USA_YR7AFQ_2021
ON311186.1_USA-WA_2021
ON311187.1_USA-WA_2021
ON311185.1_USA-WA_2021
OK649365.1_RV-B27_USA_4N4Q2D_2021
MZ629146.1_RV-B27_USA_KJTADV_2021
MZ835597.1_RV-B27_USA_PSJ3JS_2021
OK539471.1_RV-B27_USA_35PEXM_2021
ON311189.1_USA-WA_2020
ON311195.1_USA-WA_2020
ON311194.1_USA-WA_2020
MZ835570.1_USA-WA_2021
OK649400.1_RV-B27_USA_K7UHH5_2021
ON311190.1_USA-WA_2020
ON311200.1_USA-WA_2020
ON311198.1_USA-WA_2020
MZ363452.1_RV-B27_USA_RBDWWJ_2021
OK539488.1_RV-B27_USA_HBCZ2B_2021
MZ363412.1_RV-B27_USA_2DPZS9_2021
OQ533275.1_China_2023
KY369900.1_USA-WA_2016
KY369901.1_USA-WA_2016
2019.87
2019.7
2019.64
2019.43
2020.71
2019.22
2019.34
2019.23
2019.08
2018.93
2014.13
2019.27
2009.33
2011.08
2005
2010
2015
2020
2025
B70
1.7E-3 subst/site/year
95%HPD [1.4E-3, 2.0E-3]
PP194025.1_RV-B70/USA/7Z27Z7/2022
PP194029.1_RV-B70/USA/NN5YW0/2022
PP194028.1_RV-B70/USA/FKXAA3/2022
PP194026.1_RV-B70/USA/109017/2022
PP194024.1_RV-B70/USA/6VMNR5/2022
OM001422.1_RV-B70/USA/D2PNJ2/2021
OL961525.1_RV-B70/USA/EPDGQT/2021
MK501737.1_Colombia_2018
KY369903.1_USA-WA_2016
KY369902.1_USA-WA_2016
MW587080.1_China_2017
HRV-B70_JQ245974_USA_2008
MH828513.1_VietNam_2013
MW587091.1_China_2017
OQ747183.1_China_2022
2020.43
2017.95
2017.19
2015.95
2015.13
2014.53
2011.48
2011.37
2010.91
2007.35
1993.99
2007.78
2001.04
2015.12
1985
1990
1995
2000
2005
2010
2015
2020
2025
C1
1.4E-3 subst/site/year
95%HPD [1.1E-3, 1.8E-3]
MW969527.1_RV-C1/USA/69719/2021
ON311180.1_USA-WA_2020
ON311179.1_USA-WA_2021
MZ322915.1_RV-C1/USA/8HV75D/2021
OK017918.1_RV-C1/USA/82JMKJ/2021
MZ268720.1_RV-C1/USA/412768/2021
HRV-C1_EF077279_2001
HRV-C1_HQ123443_Thailand_2006
MZ670576.1_RV-C1/USA/8TWEKH/2021
OM001407.1_RV-C1/USA/9HZPGH/2021
OL133760.1_RV-C1/USA/VJGVAT/2021
OK539509.1_RV-C1/USA/WMDSED/2021
MZ268706.1_RV-C1/USA/8QSTY6/2021
MZ363446.1_USA-WA_2021
OK254829.1_RV-C1/USA/5BUGGW/2021
OM001354.1_USA-WA_2021
2019.72
2019.64
2018.85
2019.31
1986.26
1992.57
1980.49
2019.56
2018.9
2016.85
2020.94
1970
1975
1980
1985
1990
1995
2000
2005
2010
2015
2020
2025
C3
2.8E-3 subst/site/year
95%HPD [2.4E-3, 3.1E-3]
32 sequences from WA 2021
LC699425.1_Japan_2018
OK017931.1_RV-C3/USA/E6M6C4/2021
OL961537.1_USA-WA_2021
MN228693.1_USA-California_2019
KY460513.2_Taiwan_2009
HRV-C3_JN990700_USA_2009
HRV-C3_JN798567_USA-Wisconsin_1999
2015.96
2018.17
2012.57
2020.97
2014.41
2006.65
2004.7
1996.89
1995
2000
2005
2010
2015
2020
2025
C11
2.1E-3 subst/site/year
95%HPD [1.8E-3, 2.4E-3]
34 sequences from WA 2021
MN163127.1_China_2017
MZ153255.1_RV-C11/USA/2FWR4/2021
MZ268710.1_RV-C11/USA/45373/2021
MH685698.1_Uganda_2013
HRV-C11_EU840952_Switzerland_2008
OK254853.1_RV-C11/USA/EC3MTA/2021
OM001465.1_RV-C11/USA/XB3862/2021
MZ629171.1_RV-C11/USA/VXVRZL/2021
OQ348028.1_USA-WA_2022
OK017944.1_RV-C11/USA/N2VNU5/2021
MZ363458.1_RV-C11/USA/TLA26P/2021
MZ363462.1_RV-C11/USA/VDNWYA/2021
MZ153244.1_RV-C11/USA/QZWH6/2021
MZ363441.1_RV-C11/USA/CU6WWX/2021
2008.53
2015.62
2001.36
2016.63
2008.72
2004.33
1990.28
2019.99
2019.3
2016.29
2018.61
2015.05
1985
1990
1995
2000
2005
2010
2015
2020
2025
C15
3.5E-3 subst/site/year
95%HPD [3.2E-3, 3.8E-3]
11 sequences from WA 2021
ON311173.1_USA-WA_2020
MZ153254.1_RV-C15/USA/2XWQD/2021
MG881840.1_Argentina_2015
MZ427501.1_RV-C15/USA/G79LPJ/2021
MZ460707.1_RV-C15/USA/VCPFJV/2021
MZ540947.1_RV-C15/USA/PSC9G8/2021
OK017942.1_RV-C15/USA/MMYYYH/2021
PP194055.1_RV-C15/USA/090357/2022
MZ460675.1_RV-C15/USA/7LX7P3/2021
OL638433.1_RV-C15/USA/GNFRBU/2021
MZ363414.1_RV-C15/USA/5JWXTT/2021
ON311174.1_USA-WA_2020
OP342700.1_China_2014
KY348864.1_Switzerland_2009
HRV-C15_GU219984_USA_2007
6 sequences from WA 2022
MN306035.1_USA-WA_2019
MN306026.1_USA-WA_2019
MN369033.1_USA-WA_2018
OP342698.1_China_2017
HRV-C15_JF317014_China_2008
HRV-C15_JN837688_USA-Wisconsin_1999
2020.22
2019.64
2013.63
2011.57
2020.84
2017.53
2016.55
2011.24
2020.26
2018.94
2008.14
2005.43
1999.75
2017.55
2014.53
2008.53
1990.94
2004.23
1985
1990
1995
2000
2005
2010
2015
2020
2025
C17
1.6E-3 subst/site/year
95%HPD [1.0E-3, 2.1E-3]
MZ221153.1_RV-C17/USA/372634-4/2021
MZ670590.1_RV-C17/USA/RAPS7Y/2021
MZ363447.1_RV-C17/USA/KUC66R/2021
MZ363451.1_RV-C17/USA/QWW85V/2021
MZ268739.1_RV-C17/USA/VA72NU/2021
MZ460682.1_RV-C17/USA/ALZEA6/2021
OL365070.1_RV-C17/USA/X2GEJC/2021
MZ153258.1_USA-WA_2021
MZ268734.1_RV-C17/USA/PA4GYJ/2021
MZ460695.1_RV-C17/USA/LQHVSZ/2021
ON311276.1_USA-WA_2020
MZ268699.1_RV-C17/USA/255977/2021
MZ363427.1_RV-C17/USA/301397/2021
MW969526.1_RV-C17/USA/70961/2021
MZ268701.1_USA-WA_2021
MZ460692.1_RV-C17/USA/KVLY8C/2021
MZ460696.1_RV-C17/USA/LSW5PQ/2021
MZ268705.1_RV-C17/USA/7M3XTG/2021
MZ460673.1_RV-C17/USA/3V8LMU/2021
MZ363439.1_RV-C17/USA/BCNLYB/2021
MZ268712.1_RV-C17/USA/278067/2021
OK017945.1_RV-C17/USA/NAJQX5/2021
MZ268727.1_RV-C17/USA/ADYKUA/2021
MZ268730.1_RV-C17/USA/J6TJX2/2021
OK181457.1_RV-C17/USA/6KXCFQ/2021
MZ153245.1_RV-C17/USA/RCC55/2021
MZ629172.1_RV-C17/USA/WB6M3K/2021
MZ629181.1_RV-C17/USA/YLEL8X/2021
MZ268698.1_RV-C17/USA/163780/2021
MZ268736.1_USA-WA_2021
MZ268721.1_RV-C17/USA/413860/2021
ON311279.1_USA-WA_2020
10 sequences from WA 2021
PP194102.1_RV-C17/USA/386123/2022
PP194103.1_RV-C17/USA/386156/2022
PP194100.1_RV-C17/USA/101675/2022
PP194101.1_RV-C17/USA/311825/2022
OR220808.1_China_2023
MZ153277.1_RV-C17/USA/368038-4/2021
MZ153278.1_RV-C17/USA/368020-4/2021
OK161409.1_RV-C17/USA/2SH4TA/2021
HRV-C17_JN815240_USA-Wisconsin_2009
MZ667421.2_USA-WA_2009
HRV-C17_JN815244_USA-Wisconsin_2008
2020.48
2019.76
2019.46
2019.28
2018.79
2018.69
2018.62
2019.71
2018.29
2019.89
2015.77
2018.33
2015.06
2018.78
2012.4
2004.13
2020.91
2018.39
1994.45
2006.55
2001.08
1985
1990
1995
2000
2005
2010
2015
2020
2025
C42
2.9E-3 subst/site/year
95%HPD [2.3E-3, 3.4E-3]
11 sequences from WA 2021
MZ268647.1_USA-WA_2021
MZ268709.1_RV-C42/USA/44858/2021
OK649363.1_USA-WA_2021
PP187407.1_RV-C42/USA/449607/2023
MZ153246.1_RV-C42/USA/YGHJB/2021
MZ268666.1_USA-WA_2021
MZ835616.1_RV-C42/USA/XY4C8S/2021
OP342691.1_China_2014
MH330336.1_India_2013
MH752985.1_USA-California_2016
KJ675505.1_Malaysia_2010
HRV-C42_JQ994500_USA_1999
2017.84
2013.97
2020.57
2017.03
2016.33
2018.95
2009.61
1996.15
1988.39
2002.24
2008.36
1980
1985
1990
1995
2000
2005
2010
2015
2020
2025
C56
2.6E-3 subst/site/year
95%HPD [2.0E-3, 3.2E-3]
MW802583.1_RV-C56/USA/69444/2021
MZ268682.1_USA-WA_2021
ON311150.1_USA-WA_2020
MZ268726.1_RV-C56/USA/466615/2021
MZ322916.1_RV-C56/USA/9CDWUK/2021
MZ363428.1_RV-C56/USA/367598/2021
MZ363435.1_RV-C56/USA/466519/2021
MW969528.1_RV-C56/USA/69486/2021
MZ363467.1_RV-C56/USA/Y43KYC/2021
ON989186.1_USA-Arizona_2020
MW679009.1_USA-Arizona_2020
MZ153279.1_RV-C56/USA/367987-4/2021
MZ268729.1_RV-C56/USA/E5AKW2/2021
MZ629123.1_RV-C56/USA/6LBP22/2021
MW969529.1_RV-C56/USA/60602/2021
MZ268724.1_RV-C56/USA/465986/2021
MG950179.1_USA-California_2017
MN163128.1_China_2017
MG950178.1_USA-California_2016
2020.97
2019.71
2018.84
2018.74
2020.92
2018.46
2018.69
2016.7
2015.53
2017.45
2020.06
2014.84
2020.66
2015.06
2015
2020
C20
8.4E-3 subst/site/year
95%HPD [5.2E-3, 0.011]
41 sequences from WA 2022
MZ268718.1_RV-C20/USA/412252/2021
MZ268722.1_RV-C20/USA/414373/2021
ON311165.1_USA-WA_2021
ON311167.1_USA-WA_2021
KY983589.1_USA-WA_2015
2019.42
2016.23
2020.99
2020.29
2020.98
2014.32
2015
2020
