# Supplementary material for: Genomic Epidemiology and Evolution of Rhinovirus in Western Washington State, 2021–2022
Source: J Infect Dis. 2024 Jul 4;231(1):e154–64. doi: 10.1093/infdis/jiae347 (PMC11793040; doi:10.1093/infdis/jiae347)

Supplementary Figure 6. Time of the most recent common ancestor (tMRCA) of the Puget Sound RV clades detected in 2021 and 2022. The tMRCA for each high-frequency genotype is denoted with a colored circle in a temporal line (pink for RV-A genotypes, green for RV-B and blue for RV-C). In addition, the 95% higher posterior density intervals are indicated with a back line.

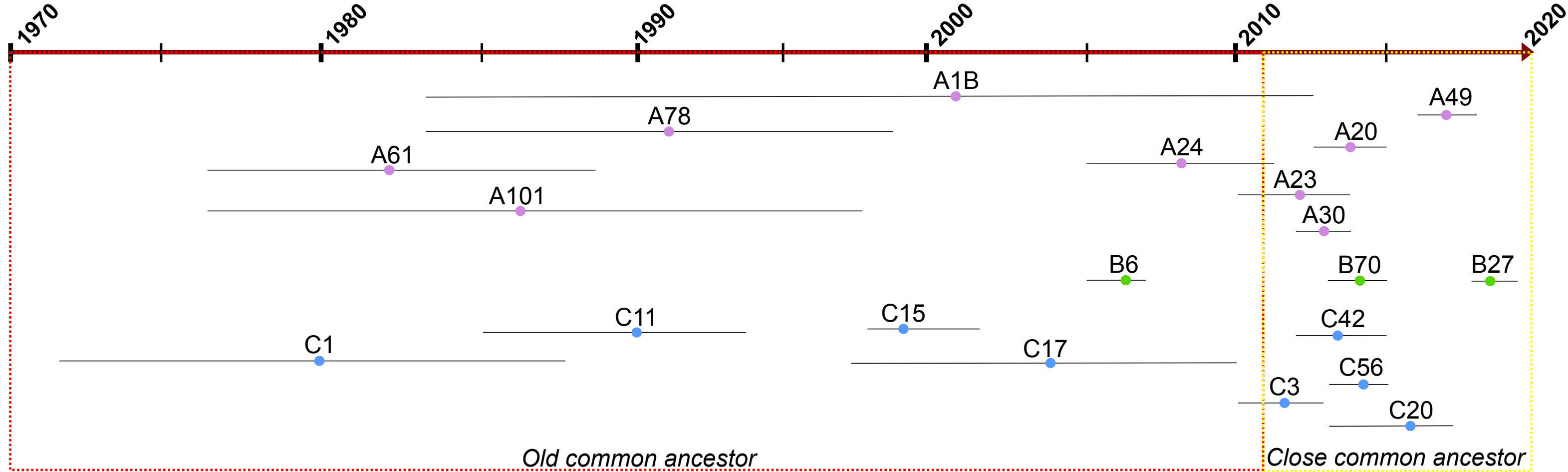

Supplement: jiae347_Supplementary_Data [file jiae347_supplementary_data.zip › SupplementaryFigure6_R1_202406.pdf]
