# Supplementary material for: Genomic Epidemiology and Evolution of Rhinovirus in Western Washington State, 2021–2022
Source: J Infect Dis. 2024 Jul 4;231(1):e154–64. doi: 10.1093/infdis/jiae347 (PMC11793040; doi:10.1093/infdis/jiae347)
Supplement: jiae347_Supplementary_Data [file jiae347_supplementary_data.zip › SupplementaryFigure7_R1_202406.pdf]

Supplementary Figure 7. Shannon entropy of the RV polyprotein sequence for prevalent genotypes. Genomes from 2021 and 2022 in Washington State from the same genotype were aligned, trimmed according to the polyprotein open reading frame, and translated with the universal genetic code. Genomes with nucleotide ambiguities were not included. Bar plots show the Shannon entropy value of each position of the entire polyprotein. Columns denote Shannon entropy values colored by genotype. Red arrows highligth entropy values above 1, which are considered moderate variable positions. At the botom of the figure the location of the mature proteins is shown, higlighting in yellow the capsid proteins and in violet the non-structural proteins. The number of sequences analyzed for each genotype is indicated in the legend.

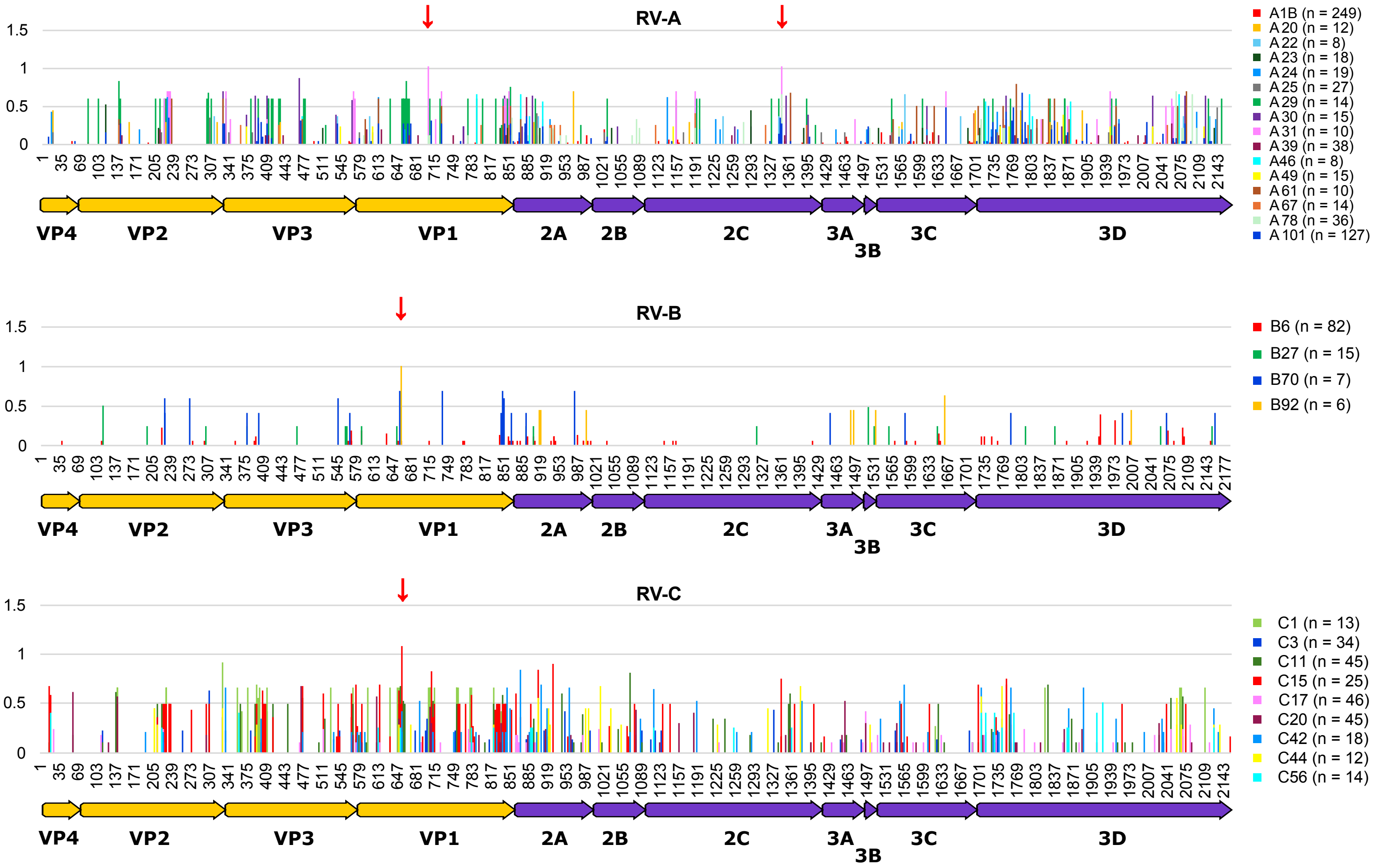

Average Shannon entropy per genotype in capsid and non structural proteins

	A1B	A20	A22	A23	A24	A25	A29	A30	A31	A39	A46	A49	A61	A67	A78
Capsid	0.0017	0.0018	0.0013	0.0027	0.0027	0.0017	0.0367	0.0070	0.0122	0.0024	0.0012	0.0009	0.0050	0.0018	0.0014
Non-structural	0.0034	0.0032	0.0022	0.0020	0.0040	0.0025	0.0091	0.0050	0.0067	0.0026	0.0121	0.0016	0.0074	0.0030	0.0052

	A101	B6	B27	B70	B92	C1	C3	C11	C15	C17	C20	C42	C44	C56
Capsid	0.0072	0.0025	0.0032	0.0075	0.0012	0.0232	0.0047	0.0188	0.0316	0.0032	0.0059	0.0080	0.0033	0.0025
Non-structural	0.0073	0.0034	0.0022	0.0080	0.0029	0.0022	0.0030	0.0086	0.0117	0.0042	0.0053	0.0099	0.0087	0.0037
