# Supplementary material for: Genomic Epidemiology and Evolution of Rhinovirus in Western Washington State, 2021–2022
Source: J Infect Dis. 2024 Jul 4;231(1):e154–64. doi: 10.1093/infdis/jiae347 (PMC11793040; doi:10.1093/infdis/jiae347)
Supplement: jiae347_Supplementary_Data [file jiae347_supplementary_data.zip › SupplementaryFigure8_R1_202406.pdf]

Supplementary Figure 8. Rhinovirus genomes available in NCBI GenBank after our study in Puget Sound region, WA.
Bar plots show the cumulative number of genomes of each RV genotype available in NCBI after our study in Puget Sound region (database reviewed in September 2023). In yellow the number of genomes per genotype before our study is indicated and in green the number of genomes per genotype we have sequenced and publicly shared is highlighted. The new genotypes described in this study are written in red.

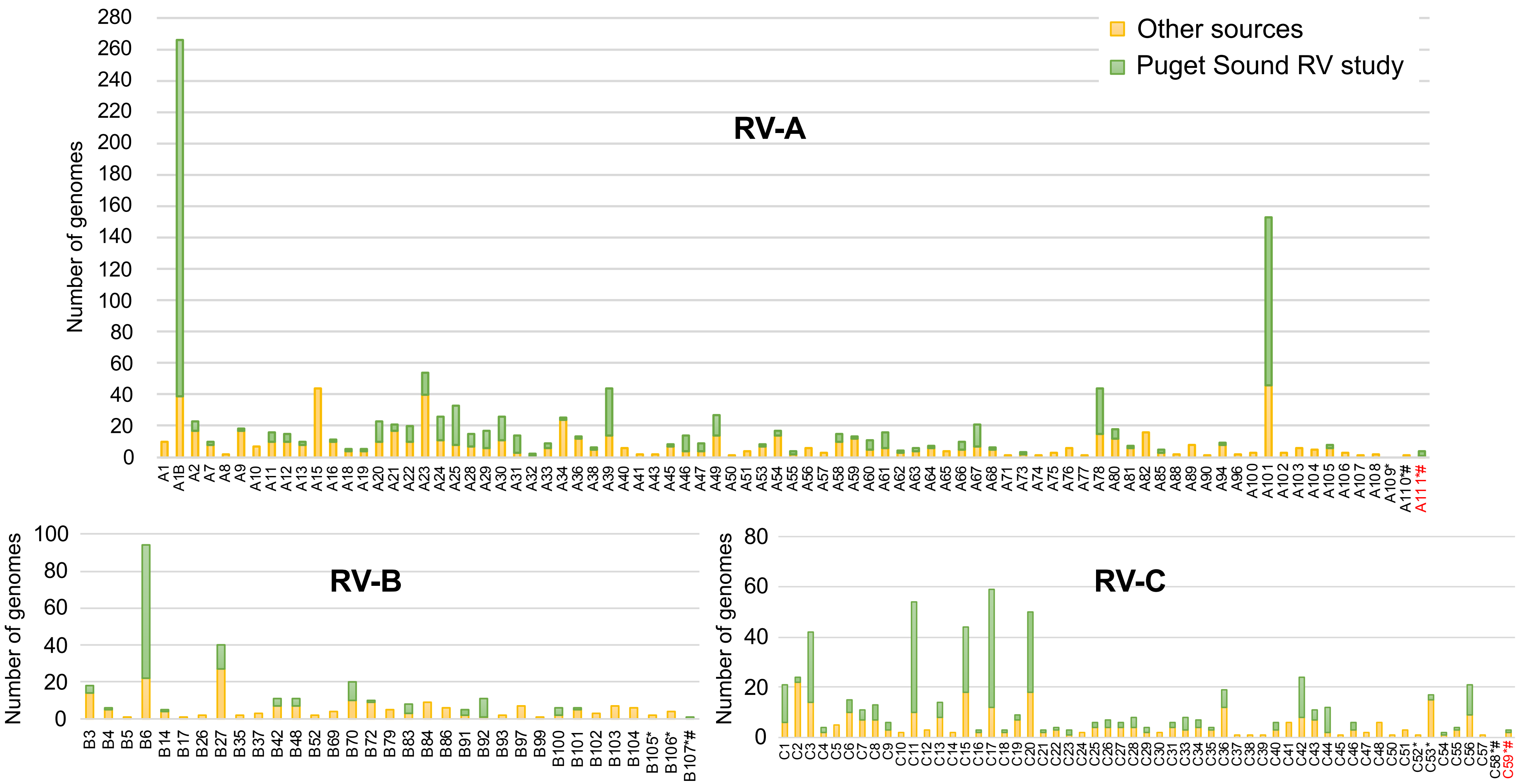

* genotype not confirmed in ICTV (https://ictv.global/report/chapter/picornaviridae/picornaviridae/enterovirus)
# genotype not confirmed by the Picornaviridae Study Group (https://www.picornastudygroup.com/)
