# Supplementary material for: Genomic Epidemiology and Evolution of Rhinovirus in Western Washington State, 2021–2022
Source: J Infect Dis. 2024 Jul 4;231(1):e154–64. doi: 10.1093/infdis/jiae347 (PMC11793040; doi:10.1093/infdis/jiae347)

**Supplementary Figure 1. Seasonality of respiratory viruses in symptomatic patients in Washington State from 2019 to 2023.** The positivity rate per month is detailed for different respiratory viruses. Rhinovirus is highlighted with red line including triangles. Grey shadow at the background of the plot informs the number of screened samples per month. The two studied periods of this work are denoted with dotted squares (P1= period 1, P2=period 2).

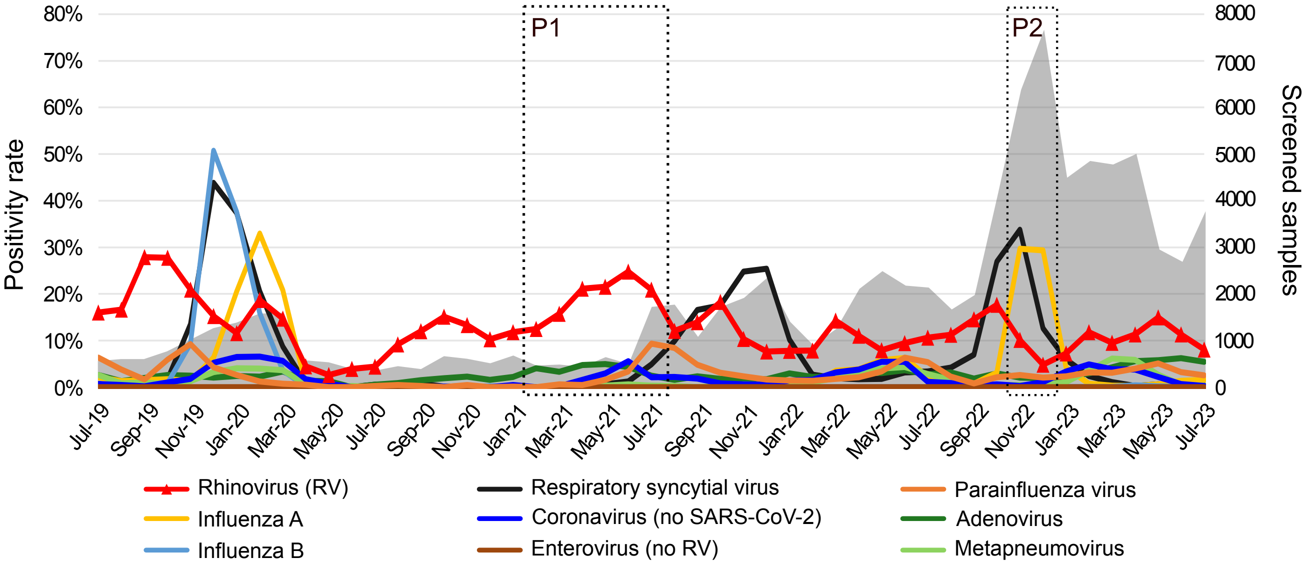

Supplement: jiae347_Supplementary_Data [file SupplementaryFigure1_R1_202405.pdf › SupplementaryFigure1_R1_202405.pdf]
